# Cordycepin generally inhibits growth factor signal transduction in a systems pharmacology study

**DOI:** 10.1002/1873-3468.15046

**Published:** 2024-11-07

**Authors:** Steven Lawrence, Jialiang Lin, Asma Khurshid, Wahyu Utami, Richa Singhania, Sadaf Ashraf, Graeme J. Thorn, Irengbam Rocky Mangangcha, Keith Spriggs, Dong‐Hyun Kim, David Barrett, Cornelia H. de Moor

**Affiliations:** ^1^ School of Pharmacy, Biodiscovery Institute University of Nottingham UK; ^2^ Present address: DOW Research Institute of Biotechnology and Biomedical Sciences DOW University of Health Sciences Karachi Pakistan; ^3^ Present address: Faculty of Pharmacy Universitas Muhammadiyah Surakarta Surakarta Indonesia; ^4^ Present address: Department of Neurology Weill Cornell Medicine New York NY USA; ^5^ Present address: Medway School of Pharmacy Universities of Kent and Greenwich Chatham UK; ^6^ Present address: Centre for Biomarkers and Biotherapeutics Barts Cancer Institute, Queen Mary University of London London UK; ^7^ Present address: Department of Zoology, Deshbandhu College University of Delhi New Delhi India

**Keywords:** cancer, cordycepin, ERK, mTOR, PI3K, signal transduction

## Abstract

Cordycepin (3′ deoxyadenosine) has been widely researched as a potential cancer therapy, but many diverse mechanisms of action have been proposed. Here, we confirm that cordycepin triphosphate is likely to be the active metabolite of cordycepin and that it consistently represses growth factor‐induced gene expression. Bioinformatic analysis, quantitative PCR and western blotting confirmed that cordycepin blocks the PI3K/AKT/mTOR and/or MEK/ERK pathways in six cell lines and that AMPK activation is not required. The effects of cordycepin on translation through mTOR pathway repression were detectable within 30 min, indicating a rapid process. These data therefore indicate that cordycepin has a universal mechanism of action, acting as cordycepin triphosphate on an as yet unknown target molecule involved in growth factor signalling.

## Graphical abstract

Cordycepin is a natural product from the fungus *Cordyceps militaris*, a well‐known Chinese health food. Cordycepin has promise for diseases from osteoarthritis to cancer, but how it works was unclear. In systems pharmacology studies, short treatments with cordycepin blocked growth factor signalling, repressing the PI3K/AKT/mTOR and ERK pathways in 6 cell lines. Our findings explain the anticancer effects of cordycepin.

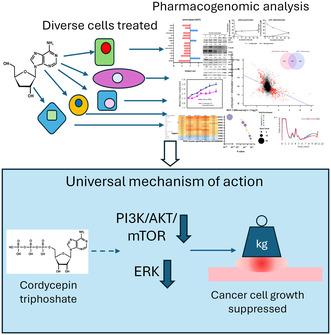

## Abbreviations

**4EBP**, translation initiation factor eIF4E binding protein, encoded by the EIF4EBP1 and EIF4EBP2 genes

**AKT**, protein kinase B, encoded by three genes AKT1, AKT2, and AKT3

**AMPK**, adenosine monophosphate‐activated kinase

**ATP**, adenosine triphosphate

**DKO**, double knockout

**DMEM**, Dulbecco's modified Eagle's medium

**DMSO**, dimethylsulfoxide

**EDTA**, ethylenediaminetetraacetic acid

**EGF**, epidermal growth factor

**ERK**, extracellular signal‐regulated kinases, encoded by MAPK1 and MAPK1

**HRP**, horseradish peroxidase

**MEK**, MAPK/ERK kinase, encoded by MEK1 and MEK2

**mTOR**, mechanistic target of rapamycin. A kinase that participates in multiple complexes, encoded by the MTOR gene

**PBS**, phosphate buffered saline

**PI3K**, phosphatidyl inositol 3‐kinase, a protein complex

**qPCR**, quantitative PCR

**RT**, reverse transcription

**TCA**, trichloroacetic acid

**WT**, wild‐type

Cordycepin (3′ deoxyadenosine) is a natural product isolated from caterpillar fungi. A large body of literature indicates it has potential as lead compound for cancer therapy and anti‐inflammatory medicines [1]. Cordycepin triphosphate has been reported to be an active metabolite of cordycepin [2, 3, 4, 5, 6] and is a known chain terminator for polyadenylation in cell‐free assays and cell culture [3, 5, 7, 8, 9, 10]. It is erroneously stated in several recent reviews that cordycepin affects transcription in general [11, 12]. However, it has been shown not to be a chain terminator for total pre‐mRNA (hnRNA) synthesis in intact cells, although it does inhibit transcription of specific mRNAs and ribosomal RNAs [3, 4, 10, 13, 14]. In addition, cordycepin has been reported to act on adenosine receptors [15, 16, 17, 18]. It is however unclear how these activities of cordycepin lead to its therapeutic effects.

In a recently performed systematic review of the biological effects of cordycepin, we noted that the literature indicates that cordycepin consistently inhibits activation of ERK MAP kinases, represses phosphatidyl inositol 3‐phosphate kinase (PI3K) signalling and activates adenosine monophosphate‐activated kinase (AMPK) [1]. High‐throughput studies of the effect of cordycepin on gene expression have been published, but most used very high doses or featured incubation times that were very long, making them less useful to deduce the direct effects of cordycepin on the proliferation and survival of cells [19, 20, 21, 22, 23, 24, 25, 26, 27, 28]. Each of these studies came to different conclusions regarding the mechanism of action of cordycepin under their experimental conditions, suggesting that there is no universal mechanism of action of cordycepin.

The present study confirms the effects of cordycepin on growth factor signalling, cell survival, proliferation in a variety of cell lines and detect accumulation of cordycepin triphosphate. High‐throughput characterisation of gene expression in six cell lines was used to determine the effects of cordycepin on mRNA levels after 2 h. In addition, polyribosome association of mRNAs after 30 min. The data were analysed to determine which pathways are affected. Our data suggest that cordycepin suppresses growth and survival signalling pathways through reducing ERK and/or PI3K signalling in all the cell lines studied.

## Materials and methods

### Materials

#### Chemicals

Cordycepin 98% pure was purchased from Sigma Aldrich (C3394, Merck, Darmstadt, Germany) or Carbosynth (ND02930, Biosynth, Compton, Berkshire, UK). It was dissolved in dimethylsulfoxide (DMSO) at 1000× the treatment concentration. Human recombinant epidermal growth factor (EGF; PHG0314; Gibco, Thermo Fisher Scientific, Loughborough, Leicestershire, UK) was dissolved in sterile water. Adenosine deaminase inhibitor: pentostatin (2′‐deoxycoformycin, 2033; Tocris, Bio‐Techne, Abingdon, Oxfordshire, UK), PI3K inhibitors: LY294002 (HY‐10108; Insight Bio, Wembley, Middlesex, UK), pictilisib (HY‐50094; Insight Bio), alpelisib (BYL‐719, HY‐15244; Insight Bio) all dissolved in DMSO. mTor inhibitor: Torin 1 (4247; Tocris), AKT inhibitor: MK‐2206 (ABE4221; Source Bioscience, Nottingham, UK), MEK inhibitor: PD98059 (9900; Cell Signaling Technology, Leiden, Netherlands), also all dissolved in DMSO.

Antibodies against phosphorylated AKT (Ser473: #4060), total AKT (#9272), phosphorylated ERK (Thr202/Tyr204: #4370), total ERK (#4695), phosphorylated 4E‐BP1 (Thr37/46: #9459), total 4E‐BP1 (#9452), phosphorylated MEK1/2 (Ser217/221: #9154), total MEK (#9122), phosphorylated AMPKα (Thr172: #2535) and total AMPKα (#5831) were purchased from Cell Signaling Technology. Vinculin antibody (#V9131) was purchased from Sigma Aldrich (Merck, Darmstadt, Germany). Anti‐Rabbit Immunoglobulins/HRP (P0217) and Anti‐Mouse Immunoglobulin/HRP (P0447) were purchased from DAKO (Agilent, Santa Clara, CA, USA).

### Tissue culture

NIH3T3 fibroblasts were obtained from the ECACC and grown in DMEM with 2 mm l‐glutamine and 10% newborn bovine serum as recommended. MCF‐7 (CVCL_0031) cells were kindly provided by Sebastiaan Winkler from the School of Pharmacy, University of Nottingham and their identity was confirmed by DNA typing (DDC Medical) within 3 years of the work. They were cultured in DMEM supplemented with 10% (v/v) fetal bovine serum, 4–6 mm l‐glutamine, 100 units·mL^−1^ penicillin and 0.1 mg·mL^−1^ streptomycin. HEK293 (CVCL_0045) derivatives WT and DKO cells were a gift from Professor Graeme Hardie (University of Dundee) and were cultured in DMEM (with glutamine) with 10% (v/v) fetal bovine serum, 100 units·mL^−1^ penicillin and 0.1 mg·mL^−1^ streptomycin. MDA‐MB‐231 Fluc were derived from MDA‐MB‐231 (CVCL_0062) cells by insertion of a luciferase gene which enables bioluminescence imaging of the cells once transplanted *in vivo* [29, 30]. These cells were kindly provided by Anna Grabowska and Philip Clarke from the Cancer Biology Unit, School of Medicine, University of Nottingham. MDA‐MB‐468 (CVCL_0419) cells were kindly provided by Tracey Bradshaw (School of Pharmacy, University of Nottingham). Both MDA‐MB‐231 Fluc and MDA‐MB‐468 were cultured in RPMI‐1640 (Sigma) with 10% (v/v) fetal bovine serum. All cells were incubated at 37 °C in 5% CO_2_. For experiments, cells were seeded at medium confluency (typically 1.5–3 × 10^4^ cells·cm^−1^) and the first treatment (e.g. serum deprivation or cordycepin treatment) happened 24 h after seeding. The cordycepin concentration to be used was determined in pilot experiments by taking the lowest concentration at which changes in gene expression for a few genes were maximal using RT‐qPCR. All cells were regularly checked for mycoplasma using the biotool quicktest v2 (Bioquote, York, UK) and/or qPCR. All human cell lines were genotyped to affirm their identity.

For serum starvation, cells were incubated in medium with 0.5% of the appropriate serum for the indicated time. Serum stimulation was performed by adding 10% serum to the dish. EGF stimulation for MDA‐MB‐468 was at 10 and 50 μm and for HEK293 cells at 15 μm.

Live cell counting was performed using a haemocytometer with trypan blue staining (Sigma). Protein synthesis was measured by incubating the cells with ^35^S labelled methionine, followed by extraction on the plate, TCA precipitation and scintillation counting and was corrected for protein content as described previously [5].

For the scratch assay, MCF‐7 cells were grown to 80% confluency and the medium was changed to 1% serum in DMEM. After 24 h, a single scratch wound was inflicted using a pipette tip. Cells were washed the medium replaced with fresh 1% serum in DMEM with the addition of 1 mm 5‐fluorouracil to prevent proliferation and with the indicated amount of cordycepin. Wound repair was calculated by subtracting the total wound area at 16 h from this area at 0 h using imagej software [31].

### Gene expression

RNA isolation was performed using Macherey‐Nagel Nucleospin Total RNA Isolation (Dueren, Germany) or Promega ReliaPrep kits (Madison, WI, USA) using the manufacturer's instructions, except that DNAse treatments were extended to 1 h. Reverse transcription (RT) was performed using random hexamers and SuperScript III (Invitrogen, Thermo Fisher Scientific). Quantitative PCR (qPCR) was performed using the Promega GoTaq qPCR Master Mix in triplicate for each RT reaction in a Stratagene Mx3005P (Thermo Fisher Scientific) or a Qiagen Rotor‐Gene Q (Hilden, Germany). The fold change was calculated using the $2^{-\Delta \Delta C_{T}}$ method [32] using GAPDH as the reference mRNA unless indicated differently. Primers can be found in Table S1. 3′ end labelling, RNA digestion with RNAse A and T1 and poly(A) electrophoresis was performed as previously described [3]. Polysome profiling was conducted as described previously on sucrose gradients [5, 33]. The gradients were fractionated into 10 fractions. Fractions 1–5 were pooled as the subpolysomal fractions and 6–10 as the polysomal fractions. For quantification of polysome gradient fractions, *Schizosaccharomyces pombe* RNA was spiked into the fractions before RNA isolation and the yeast Act1 mRNA was used as a reference.

Microarray analysis was conducted at the MRC Toxicology Unit (Leicester) using Agilent mouse and human 60K arrays in triplicate and using single colour labelling [34], except for the polysome profiles, which were by dual colour labelling with a dye swap and in duplicate. Microarray data were analysed using limma [35].

MDA‐MB‐231 Fluc Illumina RNA‐seq was performed by DeepSeq Nottingham at 75 bp paired‐end reads in duplicate. HEK293 Illumina RNA‐Seq was performed by Azenta (Genewiz, Leipzig, Germany), paired‐end 2 × 150 bp, in triplicate, with around 30 million reads per sample. Reads were mapped to the relevant genome (Human genome; GRCh38.p13, Mouse genome, GRCm38.p6) and counted using rsubread [36, 37]. All experiments were quality checked by multidimensional scaling. This revealed that replicate 1 of the HEK293 experiment was an outlier for all conditions (Fig. S6). This led us to discard this replicate. For both microarrays and RNA‐seq, gene expression profiles were interpreted using David gene ontology [38] and Ingenuity Pathway Analysis (Qiagen) [39].

### Immunoblotting

Cell lysates for immune blotting were prepared with RIPA buffer (PBS containing 0.5% (w/v) Igepal, 0.5% (w/v) deoxycholate, 0.05% SDS, 1 mm β‐glycerophosphate and 1 mm Na_3_VO_4_, 1 mm phenylmethane sulfonyl fluoride (PMSF) in PBS) and the protein concentration determined using the Pierce Coomassie Protein Assay Kit (Thermo Fisher Scientific). Western blotting was performed following SDS/PAGE electrophoresis and electroblotting on PVDF membranes. Washing of blots was in TBST (10 mm (v/v) Tris/HCl pH 8, 150 mm sodium chloride, 0.05% (v/v) Tween 20). All blots were blocked in 5% milk powder in TBST. Antibodies (see Materials and methods) were incubated in 5% milk powder in TBST except antibodies against phosphorylated proteins which were incubated in 2% BSA in TBST. After washing, peroxidase activity was detected by chemiluminescence with an ECL kit (RPN2236; GE Healthcare, Chalfont St Giles, Buckinghamshire, UK) and imaged using an ImageQuant LAS 4000 imager (GE Healthcare).

### Mass spectrometry

Cells seeded in six well plates were washed with PBS and extracted on the plate on ice with 0.5 mL 1.25 mm EDTA in methanol (prechilled on ice) and centrifuged at 15 000 ***g*** at 4 °C for 15 min. The supernatant was evaporated in a Jouan centrifugal evaporator and re‐dissolved in 50 μL water. For media samples, 1 volume of cold 1.25 mm EDTA in methanol was added and the sample was centrifuged 15 000 ***g***, 4 °C for 15 min and the supernatant was then collected. Liquid chromatography with mass spectrometry (LC–MS) was performed using an HP 1050 HPLC system (Agilent) and a Luna C‐18 column. Elution was with a gradient of 5 mm *N*,*N*‐dimethylhexylamine (DMHA) in 95% methanol to 5 mm DMHA in 80% methanol followed by analysis on a Waters Quattro Ultima triple quadrupole mass spectrometer (Waters, Milford, MA, USA).

### Animal experiment

All animal experiments were performed at EPO GmbH, Berlin‐Buch, Germany in accordance with the ‘Guidelines for accommodation and care of laboratory animals’ by the Council of Europe. Animal study protocols were approved by respective institutional ethical commission for animal experimentation and by local authorities (LAGeSo Berlin, Germany, approval number A0452/08).

10^7^ MCF‐7 cells were inoculated subcutaneously in female NMRI nu/nu mice with oestradiol supplementation. Therapy was started at palpable tumour size after randomisation of animals by tumour volume. Fifteen animals were randomly assigned to the treatment group and 15 to the control group. 22 mg·kg^−1^ cordycepin was administered in 0.1 mL injections 2 × week. The study was terminated at the humane endpoint at study day 52. Body weight and tumour volume were determined.

## Results

### Cordycepin represses serum stimulation in NIH3T3 mouse fibroblasts

**Fig. 1:**
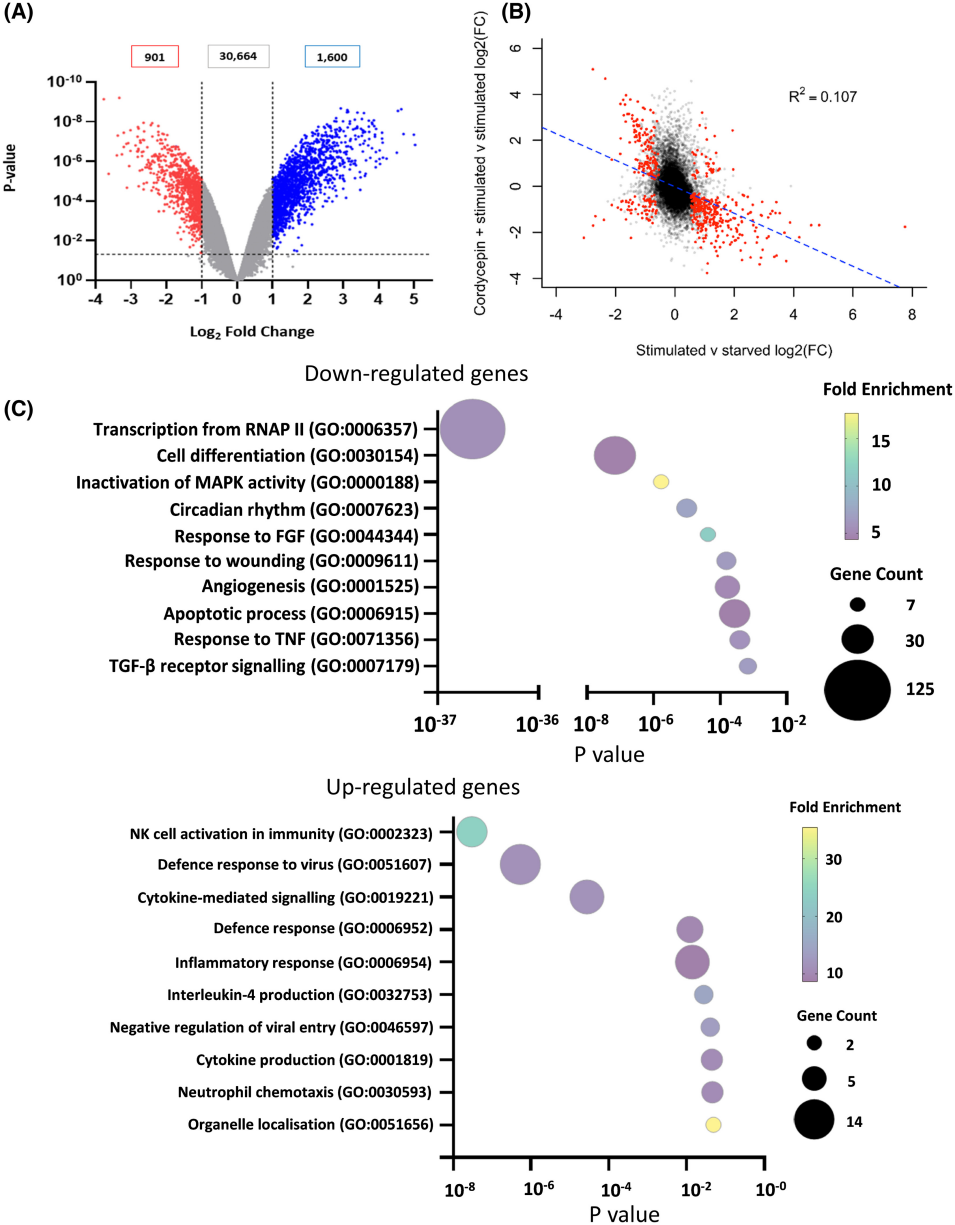
The effect of cordycepin on the serum response in a mouse fibroblast cell line (NIH3T3). NIH3T3 cells were serum starved for 24 h, treated with 20 μm cordycepin or vehicle (DMSO) for 1.5 h and then stimulated with serum for 30 min in triplicate. RNA was isolated and used for microarray analysis. (A) Volcano plot of the LogFC and the *P*‐value for the effect of cordycepin on serum‐stimulated cells. Cut‐offs of logFC ≥ 1 (blue) or LogFC < −1 (red) and *P*‐value of ≤ 0.05 are highlighted. (B) Correlation between the 2log fold change (logFC) for the effect of serum stimulation on serum‐starved cells (in duplicate) and for the effect of cordycepin on serum‐stimulated cells (triplicate). *R* ^2^ = 0.107, *F*‐statistic: 3551 on 1 and 29 656 degrees of freedom, *P*‐value: < 2.2e‐16. (C) Gene ontology term enrichment of the top 10 GO Terms (BP).

We have previously shown that cordycepin reduces cell proliferation, inhibits mTOR signalling and activates the AMPK pathway in NIH3T3 fibroblasts [5]. This suggested that cordycepin has anti‐proliferative properties through effects on signal transduction. In order to examine the effect of cordycepin on growth factor stimulation, serum‐starved NIH3T3 cells were treated with serum in the presence and absence of 20 μm cordycepin and a microarray analysis was conducted. Cordycepin repressed the expression of 901 mRNAs and induced the expression of 1600 mRNAs (Fig. 1A).

To determine if cordycepin specifically blocks the growth signals mediated by serum stimulation, the effect of 30 min serum stimulation was compared with the effect of cordycepin on serum‐stimulated cells. As can be seen in Fig. 1B, most serum‐induced genes were repressed by cordycepin, while the effect on serum repressed genes was more variable. These data indicate that cordycepin is repressing the well‐known serum response genes, many of which are transcription factors [40]. Indeed, the transcription factors repressed by cordycepin include the well‐known serum response genes *Fos*, *FosB*, *Jun*, *JunB*, *Myc*, *Egr1* and *Egr2*.

Gene ontology (GO) analysis of the downregulated genes (Fig. 1C, upper panel) indeed gave regulation of transcription as the most prominent effect, with several pathways related to normal tissue repair and cancer cell survival and proliferation (response to FGF and TNF, response to wounding, angiogenesis) being affected. The downregulation of inactivators of MAPK activity is primarily due to effects on the dual specificity phosphatases (DUSPs). Circadian rhythm genes were also downregulated. Upregulated genes were primarily involved in immune responses other than inflammation (Fig. 1C, lower panel).

**Fig. 2:**
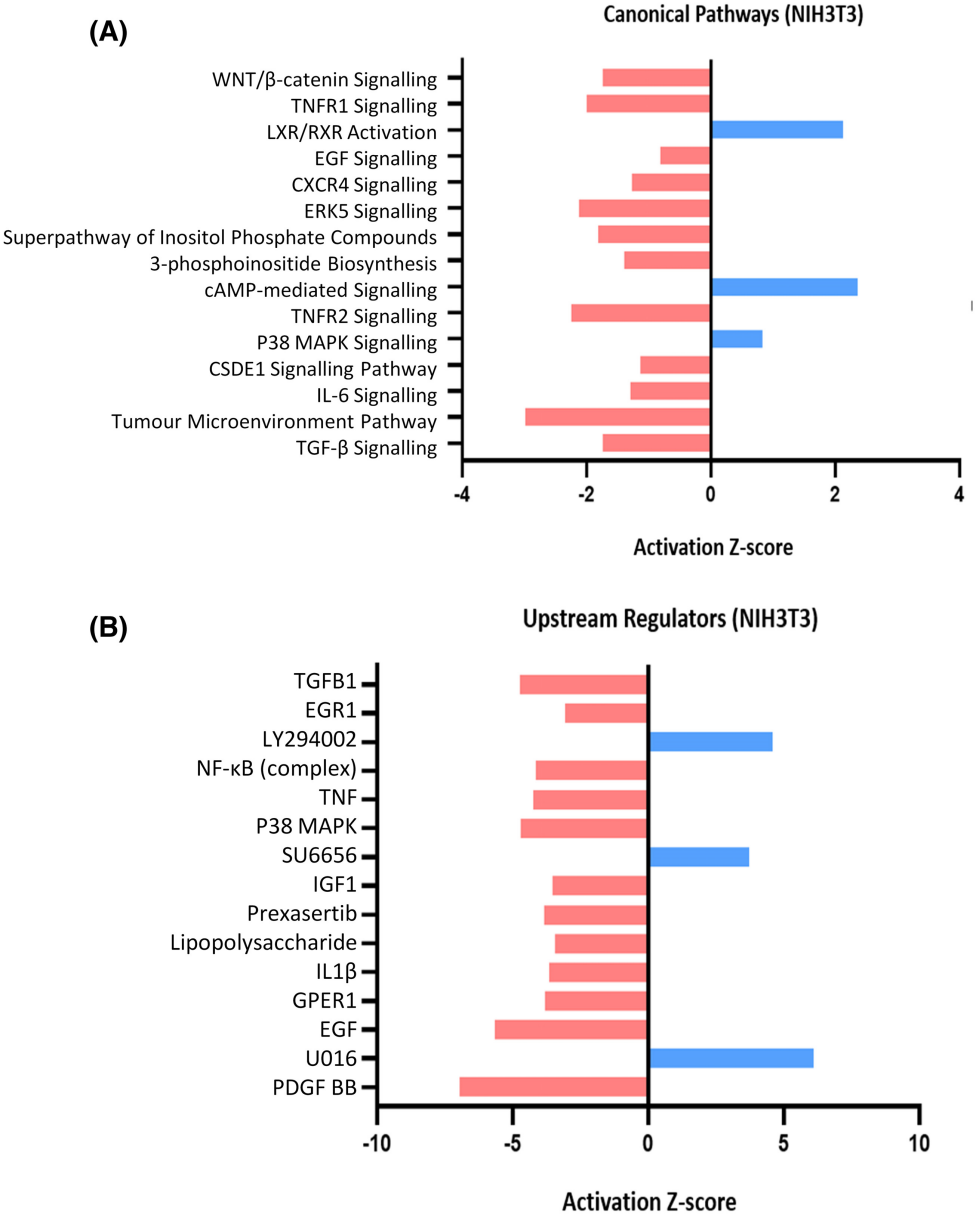
Ingenuity pathway analysis (IPA) of the effect of cordycepin on the serum response in a mouse fibroblast cell line (NIH3T3). Ingenuity pathway analysis (IPA; Qiagen) results of the data presented in Fig. 1. All statistically significant differentially expressed genes (≤ 0.05 *P*‐value from moderated *t*‐statistic) obtained through microarray output was analysed using limma [35] and entered into IPA [39]. (A) Indicates the top biological canonical pathways which are repressed (red bars) or upregulated (blue bars) with cordycepin treatment. (B) Indicates the top upstream regulators which act in an opposite way (red bars) or act similarly (blue bars) to cordycepin treatment based on activation *z*‐score.

As shown in Fig. 2, ingenuity pathway analysis (IPA) detected downregulation of many canonical pathways related to growth and cancer, including Wnt/β‐catenin, EGF signalling, TGF‐β signalling as well as repression of inflammatory pathways such as TNFR1 signalling, TNFR2 signalling and IL‐6 signalling. Notably, the Superpathway of PI3K signalling and 3′ phosphoinositide synthesis were also reduced. Upstream regulator analysis with IPA also detected downregulation of these pathways and indicated that three kinase inhibitors were predicted to have similar effects to cordycepin: the PI3K inhibitor LY294002, the Src family kinase inhibitor Y6656 and the MEK inhibitor U0126.

### Cordycepin reduces growth factor signalling in three breast cancer cell lines

**Fig. 3:**
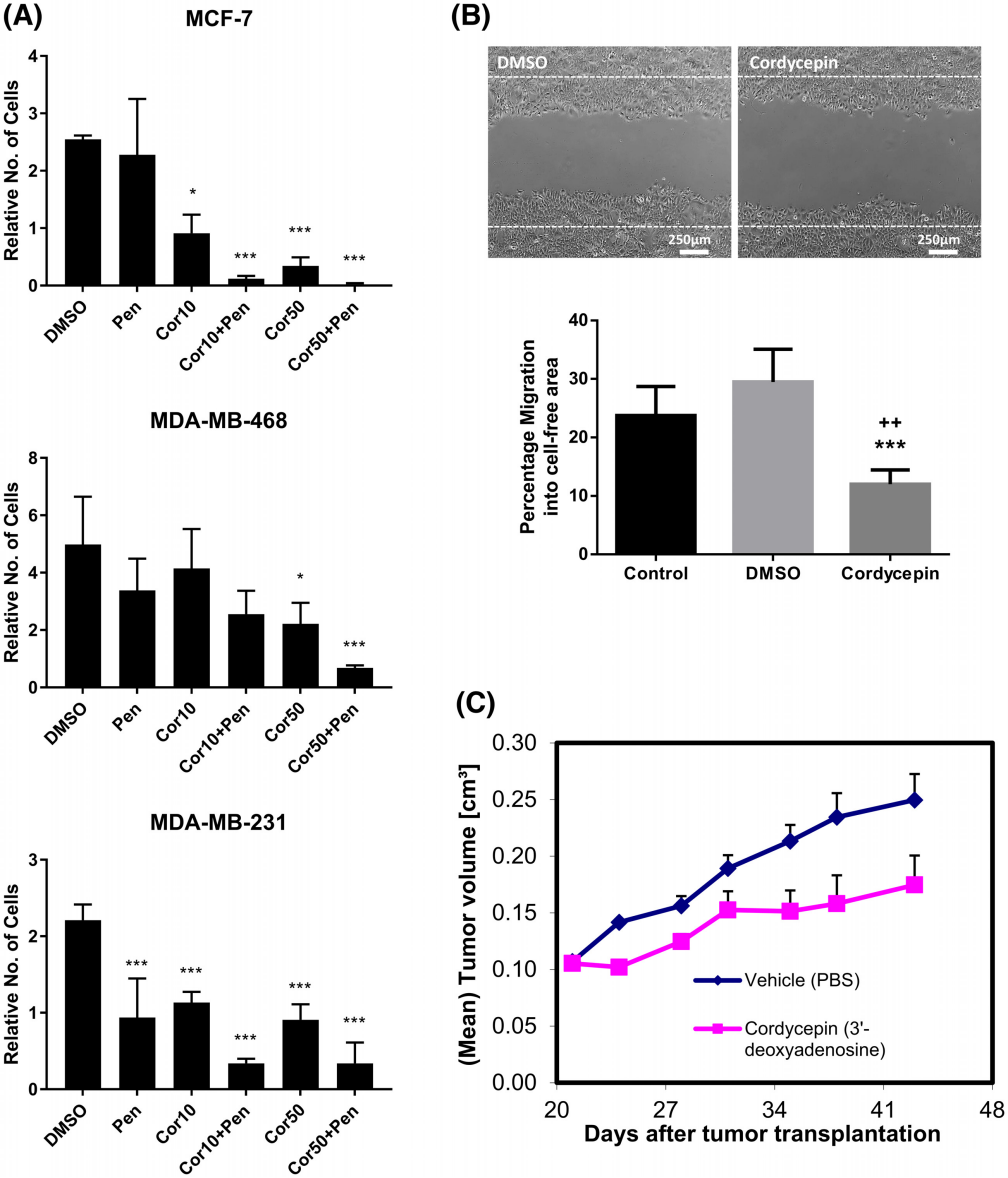
Cordycepin affects breast cancer cell proliferation and migration in tissue culture and reduces tumour growth in an animal model. Breast cancer cells were treated with vehicle (DMSO) 1 nm pentostatin (Pen) or cordycepin (Cor10 – 10 μm, Cor50 – 50 μm). (A) Live cells as determined by trypan blue stain after treatment for 72 h with cordycepin and/or pentostatin. Cells are expressed relative to the number of cells which were seeded 12 h before the start of treatment. *n* = 3, error bars are standard deviations, significance was calculated as by *t*‐test with Dunnett's correction, comparing to DMSO. *P < 0.05, ***P < 0.001. (B) Effect of 10 μm cordycepin on MCF‐7 cell migration as determined by a scratch assay after 16 h of treatment in medium with 1% serum. Control: no treatment, DMSO: vehicle control, cordycepin: cordycepin in DMSO. Error bars are SEM. ***P < 0.001 (vs DMSO), ^++^ *P* < 0.01, *n* = 4. (C) Nude mice with MCF‐7 xenografts were treated with tail vein injections of 22 mg·kg^−1^ cordycepin twice a week from 20 days after tumour transplantation. Tumour volume was monitored. Error bars are SEM. *n* = 15.

The growth signalling pathways are very popular targets in the development of cancer therapy, because mutations in these pathways are common in cancer [41]. We therefore examined the effects of cordycepin in breast cancer cell lines, in which growth factor signalling plays a large role in both oncogenesis and drug resistance [42, 43, 44, 45, 46]. MCF‐7 cells indeed have an activating mutation in PI3 kinase, which has been reported to sensitise them to the combination of repression of PI3K signalling and AMPK activation [47]. As can be seen in Fig. 3A, cordycepin reduced the cell number after a 72 h exposure to below the seeded number of cells with 50 μm (Cor50) having a larger effect than 10 μm (Cor10). This indicates that cordycepin reduces cell survival and proliferation. Addition of the adenosine deaminase inhibitor pentostatin synergised with cordycepin. The triple negative MDA‐MB‐468 and MDA‐MB‐231 cells were less sensitive to cordycepin with or without pentostatin, with the MDA‐MB‐231 cells also showing sensitivity to pentostatin alone. To examine the potential effect of cordycepin on cell migration, a scratch assay was performed on MCF‐7 cells at the lowest cordycepin dose (10 μm) for 16 h, when cell numbers were not yet affected. Cordycepin significantly reduced the migration of MCF‐7 cells (Fig. 3B). To test if cordycepin affects tumour growth *in vivo*, nude mice bearing MCF‐7 tumours were treated with twice weekly doses cordycepin (22 mg·kg^−1^). Figure 3C shows that this reduced tumour growth. These data indicate that cordycepin affects the survival, proliferation, migration and tumour growth of MCF‐7 breast cancer cells.

**Fig. 4:**
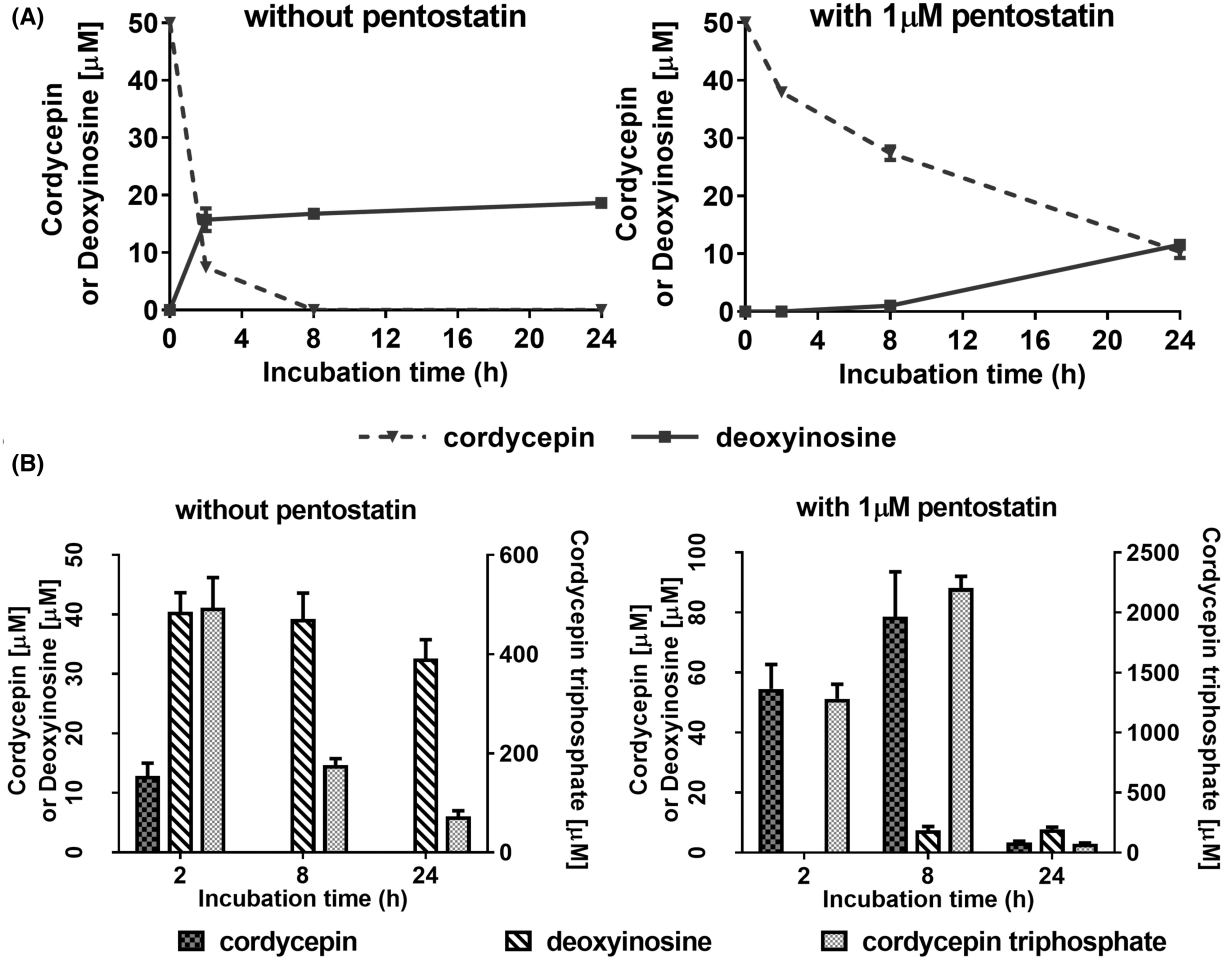
Cordycepin metabolism in medium and in MCF‐7 cells. Cordycepin, cordycepin triphosphate, deoxyinosine and adenosine phosphate levels were measured using tandem liquid chromatography and mass spectrometry in cell cultures treated with 50 μm cordycepin for the indicated times. Error bars are standard error of the mean, *n* = 6–8. (A) Concentration of cordycepin and deoxyinosine in medium over time. (B) Intracellular concentrations of cordycepin, deoxyinosine and cordycepin triphosphate. Missing bars indicate very low or undetectable levels. Note the different scale for cordycepin triphosphate on the right, *n* = 6–8.

The observed synergism between cordycepin and pentostatin suggests that deamination of cordycepin reduces its effects. To examine if deamination of cordycepin is taking place, liquid chromatography–mass spectrometry (LC–MS) was used to detect cordycepin, its deaminated metabolite 3′ deoxyinosine and its phosphorylated form, cordycepin triphosphate. As can be seen in Fig. 4A, in the absence of pentostatin, cordycepin levels in the medium dropped rapidly and 3′‐deoxyinosine levels increased. This is consistent with the reported activity of adenosine deaminases in serum [48, 49]. Pentostatin considerably slowed the deamination of cordycepin and the appearance of 3′‐deoxyinosine in the medium (Fig. 4A, right panel). Inside the cells, the predominant metabolite was cordycepin triphosphate, which was 498 μm in the absence of pentostatin at 2 h, dropping to 73 μm after 24 h (Fig. 4B, note the two different *Y* axes). In the presence of pentostatin, intracellular cordycepin triphosphate levels were highest at 8 h after treatment (2203 μm), but dropped to the same level as cordycepin alone at 24 h (73 μm). Very high levels intracellular levels of cordycepin triphosphate after cordycepin exposure have also been previously reported [50]. Unmodified cordycepin and deoxyinosine levels inside the cells were somewhat higher than in the medium in the absence of pentostatin. Pentostatin treatment increased the cordycepin levels and reduced the 3′ deoxyinosine levels as expected. These data show that cordycepin triphosphate is the major intracellular metabolite of cordycepin and that its levels are enhanced by combination treatment with pentostatin, in agreement with the effects on cell survival.

**Fig. 5:**
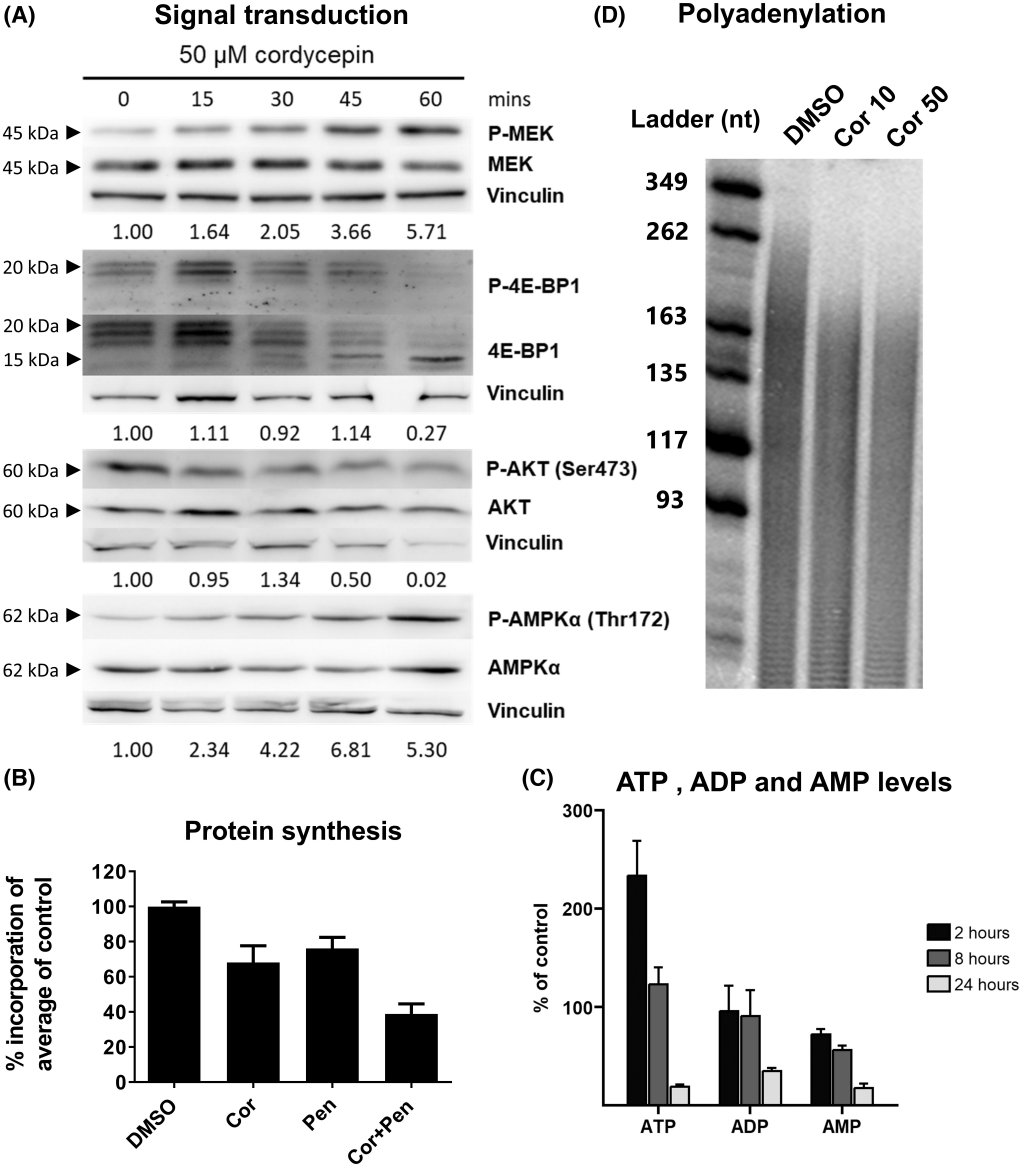
Cordycepin also affects signal transduction, polyadenylation and protein synthesis in MCF‐7 cells. (A) Western blots of cells treated with 50 μm cordycepin for the indicated times stained with the indicated antibodies. Vinculin: loading control. Numbers below the blots indicate the ratio between phosphorylated and total signal. These are representative data from three independent replicates shown in Fig. S1. (B) Effect of cordycepin on radioactive amino acid incorporation in MCF‐7 cells treated for 2 h with 50 μm cordycepin and/or 1 μm pentostatin. *n* = 3. Error bars are standard deviation. (C) ATP, ADP and AMP levels in MCF‐7 cells treated with 50 μm cordycepin over time. Error bars are standard error of the mean, *n* = 6–8. (D) Effect of cordycepin on total poly(A) tails in cells treated for 2 h.

Cordycepin has been shown to affect growth factor signalling [1]. To examine the effects of cordycepin on signal transduction in our cells, western blots were performed on MCF‐7 cells treated with 50 μm cordycepin for up to 1 h. As can be seen in Fig. 5A and Fig. S1, cordycepin induced an activating phosphorylation of MEK, in contrast with the reduction in MAPK‐dependent gene expression observed in NIH3T3 cells. However, cordycepin did reduce 4EBP and AKT phosphorylation by the mTOR kinase complexes and activated AMPK, as observed previously in other cell types by us and many others [1]. The dephosphorylation of 4EBP allows it to bind to the cap‐binding complex and inhibit protein synthesis. A reduction in the incorporation of amino acids in these cells was indeed detected (Fig. 5B), confirming the effects on this signal transduction pathway.

As AMPK is classically regulated by the AMP/ATP ratio [51], we examined whether cordycepin causes changes in adenosine nucleotides (Fig. 5C). At 2 h, when the effects of cordycepin on gene expression are very evident, ATP levels were increased and AMP levels slightly reduced, indicating that cordycepin does not activate AMPK through the classical pathway.

Cordycepin triphosphate is known to inhibit mRNA polyadenylation in nuclear extracts [7, 9] and we have previously shown that it also affects polyadenylation and 3′ end formation in primary airway smooth muscle cells [3]. To examine if cordycepin also affects polyadenylation in MCF‐7 cells, total RNA was labelled at the 3′ end and digested it with RNAses that do not cleave after adenosine. As can be seen in Fig. 5D, cordycepin reduced total cellular maximum poly(A) tail size by around 100 nucleotides at both 10 and 50 μm, indicating that cordycepin indeed affects mRNA polyadenylation in these cells.

**Fig. 6:**
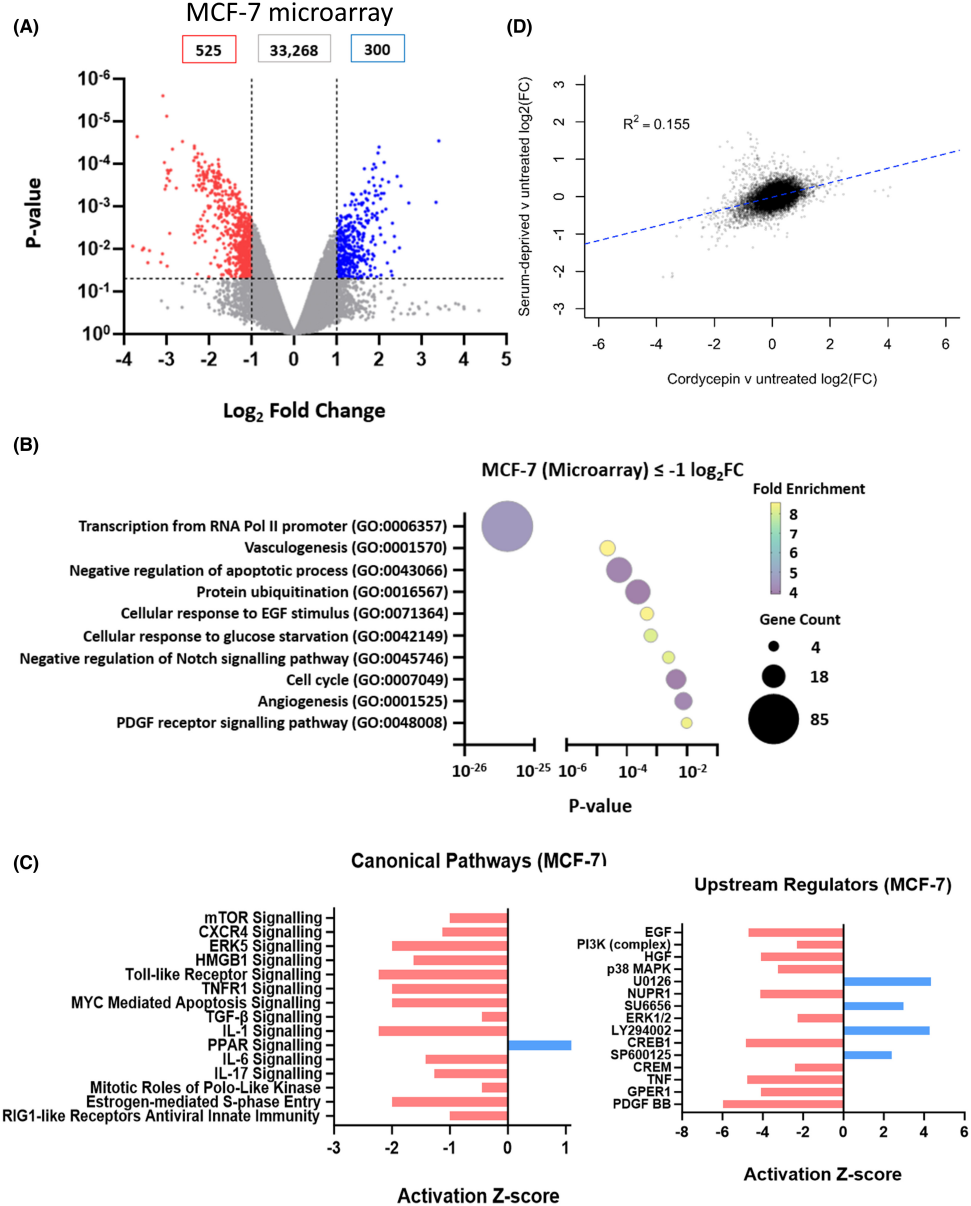
Cordycepin affects transcription regulation and genes related to growth and survival of tumours in MCF‐7 cells. MCF‐7 cells were treated with DMSO or 50 μm cordycepin for 2 h in triplicate, RNA was isolated and microarray analysis performed. (A) Volcano plot of the data as in Fig. 1. (B) Functional cluster analysis (DAVID 6.7) of downregulated genes. The same analysis for upregulated genes only yielded mRNA splicing and neuronal projection as terms. (C) Ingenuity pathway analysis of the significantly downregulated genes for canonical pathways and upstream regulators. (D) Correlation between the effect of 4 h serum deprivation and cordycepin treatment, *P* < 2.2 × 10^−16^.

To also examine the effects of cordycepin on gene expression in MCF‐7 cells, they were treated 24 h after seeding with 50 μm cordycepin for 2 h and RNA was isolated for microarray analysis. As shown in Fig. 6A, cordycepin repressed 525 genes and induced 300 genes. Downregulated genes again were enriched for transcription factors, which included cancer‐related ones such as *MYC, JUN* and *JUNB*. Indeed, the gene ontology analysis also showed many groups highly related to cancer hallmarks, such as angiogenesis, growth factor signalling by EGF and PDGF and cell cycle (Fig. 6B). Regulation of transcription was again the top term. The upregulated genes were generally poorly annotated, only giving an annotation linked to translation regulation based on the presence of two mRNAs, MCTS1 and DENR. Ingenuity pathway analysis (Fig. 6C) of the cordycepin‐induced changes predicted that many canonical pathways associated with growth factor and cytokine signalling were affected by cordycepin, including mTOR, interleukin 1 and TGF‐β. Moreover, predicted upstream regulators included EGF and PI3K and the effect of cordycepin was predicted to be similar to that of U0126 (MEK inhibitor), SU6656 (SRC inhibitor), LY294002 (PI3K inhibitor) and SP600125 (JNK inhibitor). To check if cordycepin mimics growth factor deprivation, a microarray experiment was done using RNA from control cells and cells incubated in serum‐free medium for 4 h. The two replicates proved quite variable, so no individual significant changes were detected in this experiment, but there was a significant (*P* < 2.2 × 10^−16^) positive correlation between serum deprivation and cordycepin treatment (Fig. 6D). The data suggest that cordycepin represses growth factor signals.

**Fig. 7:**
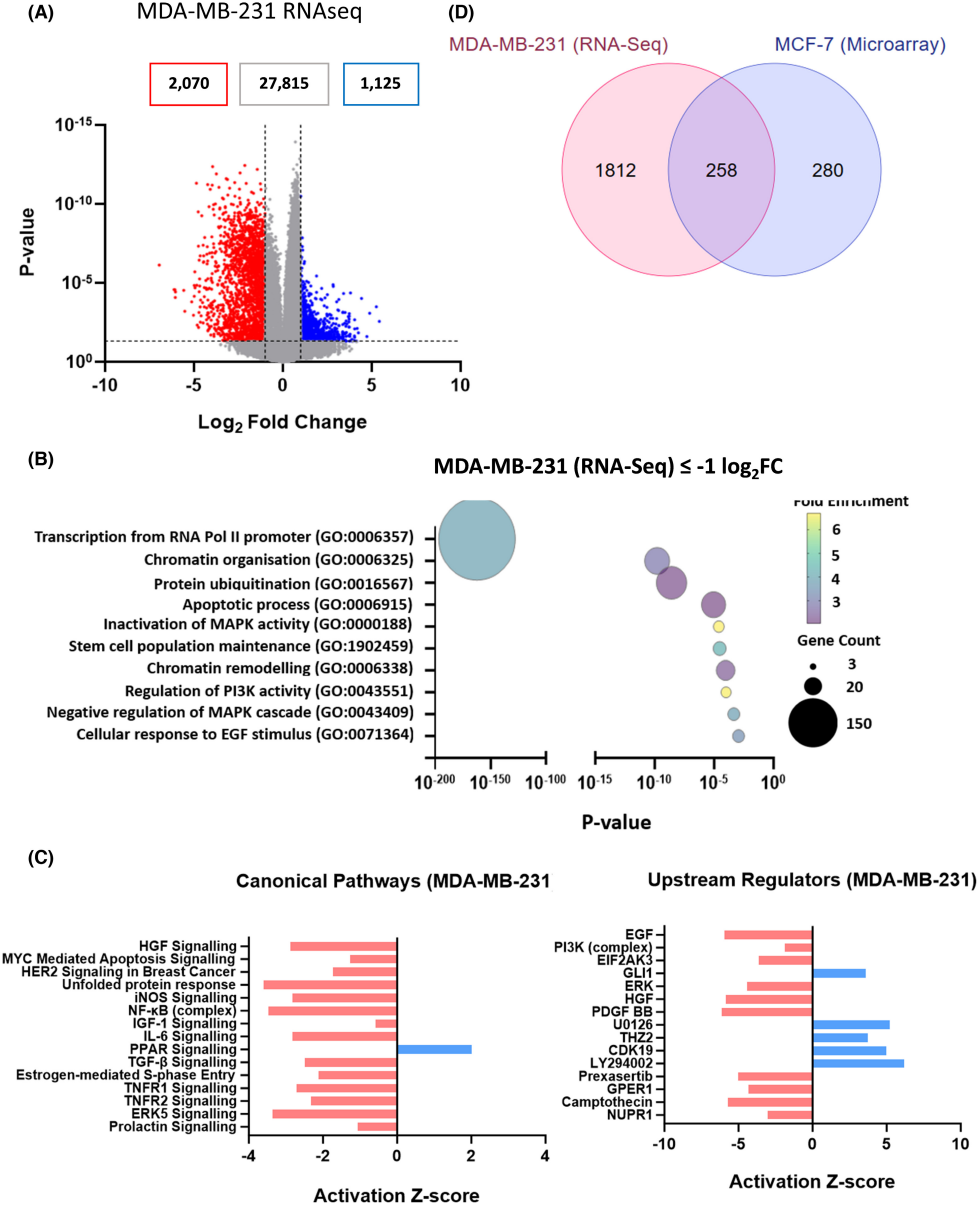
Transcription and growth factor signalling are affected by cordycepin in MDA‐MB‐231 cells. MDA‐MB‐231 Fluc cells were treated with 50 μm cordycepin or DMSO for 2 h. RNA was isolated and RNA‐seq performed in duplicate. (A) Volcano plot of the data. (B) Bubble plot indicate enriched biological pathways associated with repressed genes (red dots from A) through DAVID gene ontology. (C) Ingenuity pathway analysis of these data for canonical pathways and upstream regulators. (D) Overlap between differentially expressed genes in MCF‐7 and MDA‐MB‐231 cells.

To examine if the response of MCF‐7 cells to cordycepin is also seen in other breast cancer cells, RNA sequencing was performed for MDA‐MB‐231 treated with 50 μm cordycepin for 2 h. 2070 genes were downregulated and 1125 genes were upregulated (Fig. 7A). Figure 7B shows there was a highly significant overlap between the genes affected by cordycepin in the two cell lines (*P* < 1.44 × 10^−135^ by one‐tailed Fisher's exact test). Conversely, many other changes were cell specific, indicating that a substantial proportion of cordycepin‐induced changes is unique in these two breast cancer cell lines. However, when gene ontology analysis was performed on MDA‐MB‐231 cells, ‘Regulation of Transcription’ and ‘chromatin organisation’ were the top terms (Fig. 7C). Others included ‘Regulation of PI3K activity’ and ‘Cellular response to EGF stimulus’. Canonical pathways detected by ingenuity pathway analysis as repressed included HGF, HER2 signalling, IGF‐1 signalling, TGF‐β signalling and TNFR1/2 signalling (Fig. 7D). The top two repressed upstream regulators were again EGF and PI3K, with ERK, HGF and PDGFBB also included. Also, the effect of cordycepin was once more predicted to be similar to that of U0126 (MEK inhibitor) and LY294002 (PI3K inhibitor). These data indicate that although the exact changes in gene expression are cell type specific, cordycepin affects very similar processes in the two breast cancer cell lines.

In addition, we treated MDA‐MB‐468 breast cancer cells, which over‐express the EGF receptor, with EGF and examined the expression of specific genes that are cordycepin sensitive in MCF‐7 cells. As can be seen in Fig. S2, most of the tested genes were sensitive to cordycepin in MDA‐MB‐468 cells, including the EGF‐induced genes, such as *JUN* and *MYC*, with the exception of *DUSP1* and *AREG*. These data confirm that cordycepin can repress growth factor responses.

**Fig. 8:**
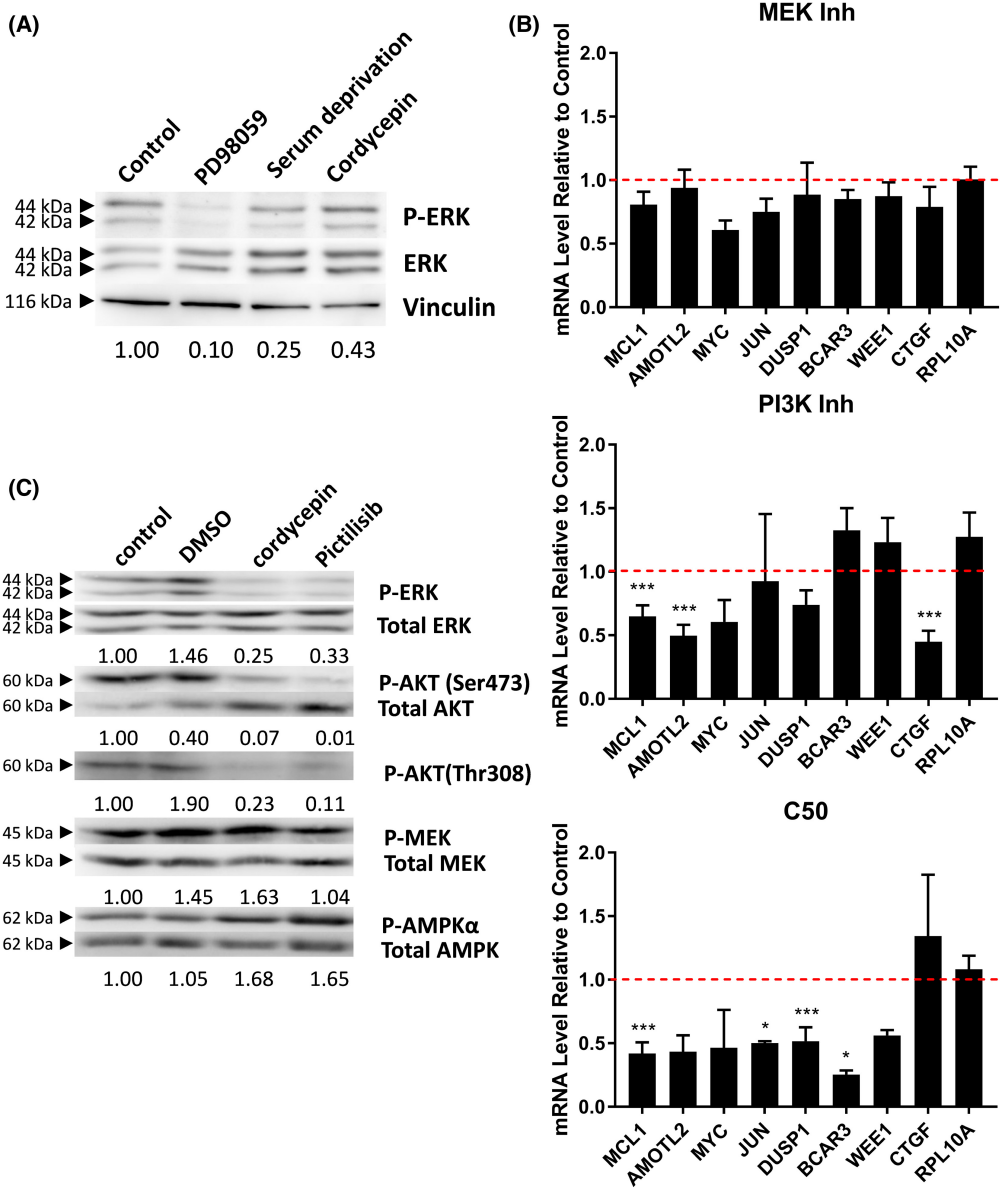
Effects of cordycepin on signal transduction and gene expression are similar to a PI3K inhibitor. (A) Western blot of lysates of MCF‐7 cells treated with 15 μm PD98059 (MEK inhibitor) for 2 h, 50 μm cordycepin for 2 h or serum starved for 4 h (no GF). Stained for phosphor‐ERK and ERK. (B) RT‐qPCR for the indicated mRNAs on RNA from cells treated with MEK inhibitor, cordycepin or PI3K inhibitor (see below). RPL10A is serving as a control gene. *n* = 3. Significance was calculated as by *t*‐test with Dunnett's correction, comparing to DMSO. *P < 0.05, ***P < 0.001. (C) Western of lysates of MCF‐7 cells treated with DMSO, cordycepin or 500 nm pictilisib (PI3 kinase inhibitor) for 2 h.

To examine if the effects of cordycepin on gene expression can be attributed to changes in signal transduction, treatments with the MEK inhibitor PD98059 and the PI3 kinase inhibitor pictilisib were compared to cordycepin application in MCF‐7 cells. As can be seen in Fig. 8A, the MEK inhibitor was much more effective in reducing the phosphorylation of ERK than cordycepin, with serum deprivation being intermediate. Despite this clear reduction in MAPK signalling, very little effect on the gene expression of cordycepin‐sensitive genes was observed (Fig. 8B). In contrast, the PI3K inhibitor pictilisib affected many of the genes repressed by cordycepin, albeit to a lesser degree. Pictilisib repressed phosphorylation of AKT on both the mTOR (Ser473) and the PDK1 (Thr308) site, as well as 4EBP to a similar extent as cordycepin and also had similar effects on ERK and AMPK phosphorylation (Fig. 8B,C). A study on the effect of a larger panel of kinase inhibitors on the expression of selected cordycepin‐sensitive genes in MCF7 cells also indicated that the effects of PI3K inhibitors were most similar to cordycepin (Fig. S3). Our data therefore suggest that the effects of cordycepin on gene expression are predominantly mediated through its effects on signal transduction by the PI3K/mTOR pathway in MCF‐7 cells.

### The therapeutic effects of cordycepin are not through activation of AMPK

**Fig. 9:**
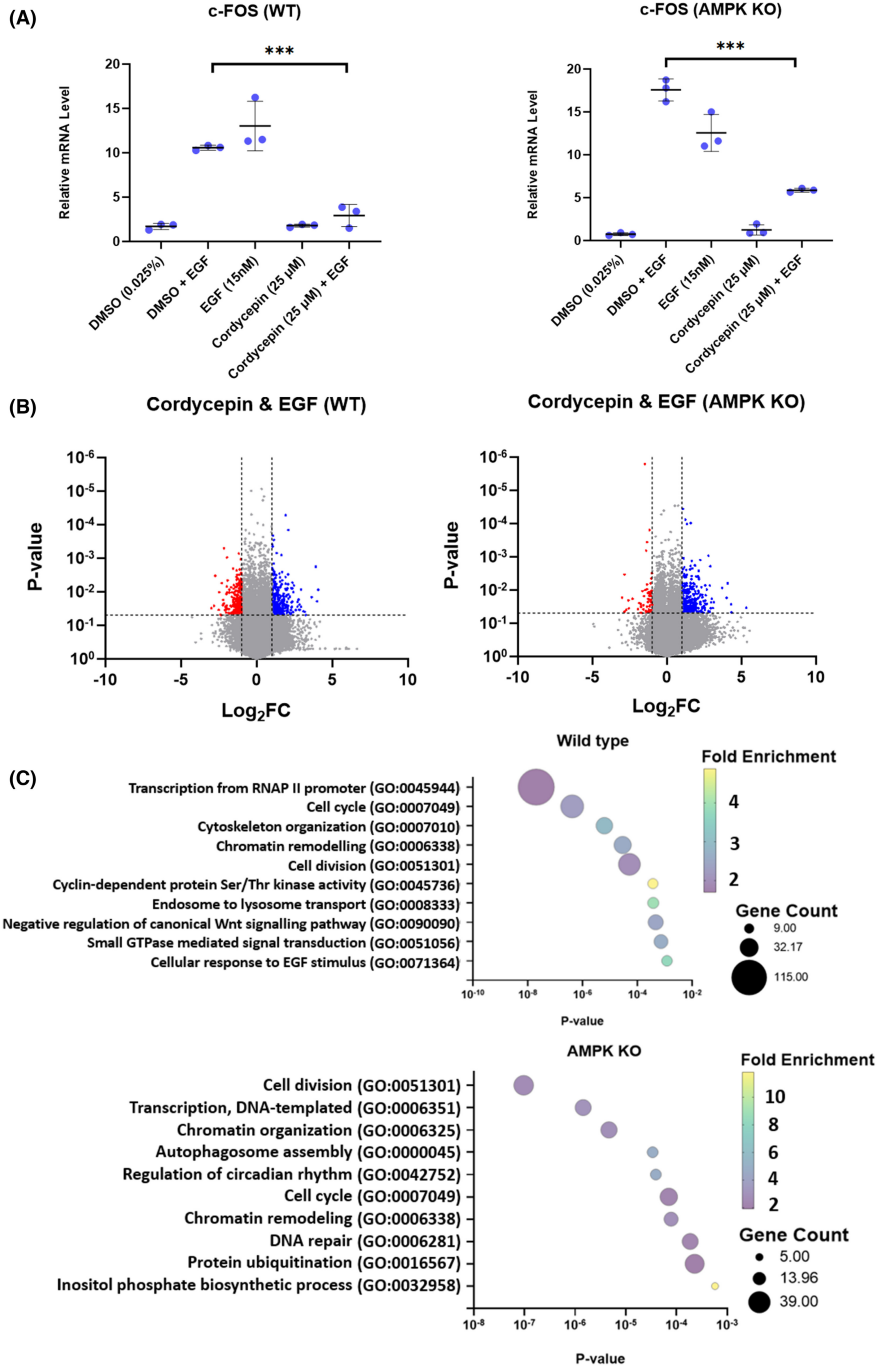
AMPK is not required for the effects of cordycepin on growth factor‐dependent gene expression in HEK293 cells. Wild‐type and AMPKα double knockout cells were serum starved 24 h prior to treatment with DMSO or 25 μm cordycepin (in DMSO) for 20 min before stimulation with EGF (15 nm) for 30 min. Total RNA was isolated. (A) RT‐qPCR for FOS. Significance was calculated as by *t*‐test with Dunnett's correction, comparing to DMSO. *n* = 3. ***P < 0.001. (B) Volcano plots of RNA‐seq of two replicates. Each dot represents a differentially expressed gene with cordycepin treatment. Red denotes genes with ≤ −1 Log_2_FC and ≤ 0.05 *P*‐value, blue denotes genes with ≥ 1 Log_2_FC and ≤ 0.05 *P*‐value, grey denotes genes with −1 to 1 Log_2_FC and > 0.05 *P*‐value. (C) Gene ontology analysis of the effect of cordycepin on EGF stimulated wild‐type (WT) and AMPK knockout HEK293 cells.

Although AMPK activation by cordycepin is very consistent across the literature [1], this pathway did not appear in the analysis of any of our high‐throughput screens. Moreover, like many kinase inhibitors, an AMPK activator did not mimic the effects of cordycepin on gene expression (Fig. S3). To definitively investigate the potential role of AMPK activation in the effect of cordycepin on growth factor signalling, HEK293 double knockout (DKO) cells were used, in which both the AMPK catalytic subunit genes have been knocked out using CRISPR [50]. We selected HEK293 cells because they are known to respond to EGF [52, 53]. Indeed, EGF induced the *FOS* gene in both WT and DKO cells, although the response was attenuated in the DKO cells. Moreover, cordycepin reduced the induction of *FOS* in both cell lines (Fig. 9A). RNA‐seq analysis of the same experiment was performed. EGF stimulation led to the induction of growth‐related mRNAs in both control and DKO cells, although the response was not identical in the two cell lines (Fig. S4). Importantly, cordycepin still had significant effects on gene expression and repressed genes related to transcription, chromatin remodelling, cell division and cell cycle, indicating that the effects of cordycepin have not been abrogated by the removal of AMPK signalling (Fig. 9B,C).

### Cordycepin affects the translation of TOP mRNAs within 30 min

**Fig. 10:**
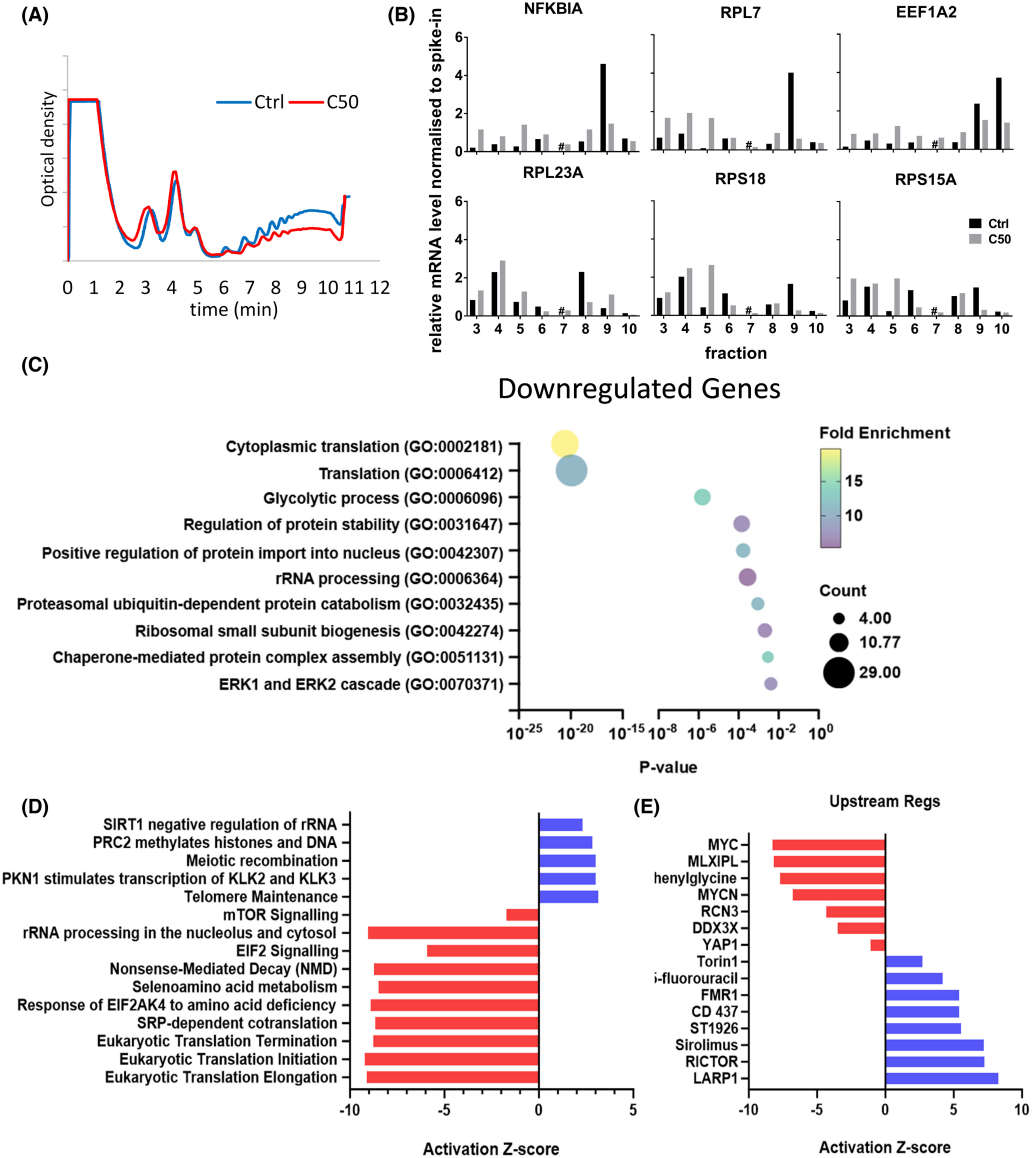
Effects of cordycepin are rapid and characteristic of inhibition of PI3K/mTOR. Polysome profiling of lysates from MCF‐7 cells treated for 30 min with DMSO (control) or 50 μm cordycepin. (A) OD at 254 nm of the sucrose gradients of replicate 1. Fractions taken every minute. (B) Relative mRNA level of selected genes in fractions 3–10 from the first replicate were measured with RT‐qPCR and presented as the ratio to the level in DMSO control. *C*_t_ values were analysed using the $2^{-\Delta \Delta C_{t}}$ method and normalised to the *C*_t_ values of spiked in budding yeast Act1. Control level is coloured in black and cordycepin treated in grey. (C) Gene ontology analysis of duplicate microarray experiment comparing pooled polysome fractions 1–5 (untranslated) and 6–10 (translated) for the downregulated genes using FC < −0.9 and *P* < 0.05. (D) Ingenuity pathway analysis for canonical pathways for this dataset. (E) Ingenuity pathway analysis for upstream regulators for this dataset.

Because all our analyses with cordycepin were 2 h after addition, there is a possibility that at least some of the effects observed are secondary changes due to the reprogramming of transcription. It was therefore decided to investigate how early effects of cordycepin can be detected. Many attempts were made to investigate when the signal transduction changes start to happen, but western blotting proved insufficiently sensitive to consistently detect the earliest changes (Fig. 5A, Fig. S1). Instead, the effect of cordycepin on PI3K/mTOR signalling was determined by examining its well‐characterised effects on translation. Polysome profiling of MCF‐7 cells was performed [5, 33]. Cells were treated with vehicle or cordycepin (50 μm) for 30 min and lysed. The lysate was fractionated by ultracentrifugation on sucrose gradients, separating mRNAs based on the number of ribosomes bound. As can be seen in Fig. 10A, mRNAs with multiple ribosomes bound (polyribosomes) were reduced, while the fractions containing one or two ribosomes (subpolysomes) were increased, confirming the effect of cordycepin on protein synthesis noted in Fig. 5B. RNA was isolated from 10 fractions of this gradient and pooled into subpolysomal (fraction 1–5) and polysomal (fraction 6–10). These RNA preparations were analysed by microarray. As can be seen in Fig. 10B, ribosomal proteins figured were heavily enriched in the subpolysomal fraction after cordycepin treatment, demonstrating that there is downregulation of the translation of terminal oligo pyrimidine tract (TOP) mRNAs, a well‐known effect of mTOR inhibition [54]. The group of mRNAs that became enriched in the polysomes upon cordycepin treatment had a less clear annotation that included steroid hormone signalling (Fig. S5). The data for downregulated ribosome association clearly demonstrate that cordycepin already affects mTOR signalling after 30 min of exposure, too early for secondary changes in transcription to cause these effects.

### Meta‐analysis of high‐throughput datasets indicates cordycepin acts on growth factor signalling

**Fig. 11:**
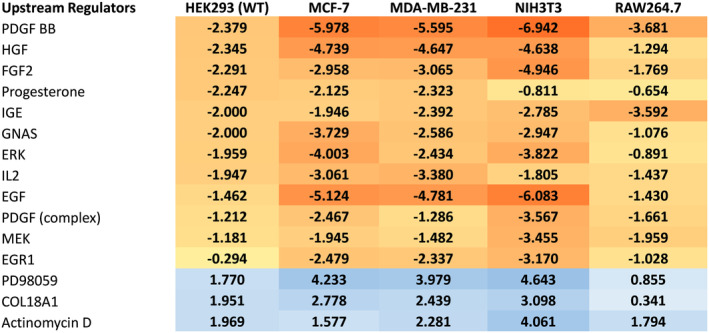
A meta‐analysis of the effect of cordycepin on five cell lines indicates it represses the effects of growth factors. Data from the experiments described above, as well as our previously published microarray data on the effect of cordycepin in RAW264.7 cells [4] were analysed using the ingenuity pathway analysis software for upstream regulators. The activation scores for this analysis are shown and negative scores are highlighted in yellow/orange, positive scores in blue.

Finally, a meta‐analysis was conducted of the analyses of the effect of cordycepin on gene expression on our datasets, including a previously published study on RAW264.9 macrophages [4]. As can be seen in Fig. 11, ingenuity pathway analysis for upstream regulators highlighted inhibition of a large number of growth factors, including PDGF, HGF and EGF. Moreover, ERK and MEK were negatively correlated, while the MEK inhibitor PD98059 positively correlated with the effects of cordycepin treatment, indicating repression of the MEK/ERK growth factor signalling pathway.

## Discussion

The data presented here demonstrate beyond reasonable doubt that cordycepin represses growth factor signalling, notably PI3K/mTOR and MEK/ERK signalling, although they do not yet provide a detailed mechanism of action. The effects of cordycepin have been remarkably consistent in our laboratory, as the experiments presented here were conducted by many researchers over a span of 15 years in a large number of cell types. Moreover, our experimental results, including the western blot analysis (Fig. 5, Fig. S1), are in agreement with those highlighted in our systematic review of the literature [1]. We also show here that cordycepin directly affects growth factor stimulation (Figs 1 and 6D, Fig. S2). The combination of the literature and the data presented here thus indicate that the mechanism of action of cordycepin is through the repression of the PI3K/AKT/mTOR and/or MEK/ERK signalling pathways in all or most cell types, suggesting that there is a universal pathway through which cordycepin has its therapeutic effects.

The data presented here clearly indicate that AMPK is not a direct target of cordycepin or required for the cellular response for cordycepin, despite the fact that cordycepin often activates this kinase [1], as also shown in Fig. 5A and Fig. S1. No changes in AMP levels in response to cordycepin were detected (Fig. 5C). No correlation of the response to cordycepin with AMPK activators or inhibitors was found by bioinformatic analysis (Figs 2, 6 and 7) and cells with an AMPK knockout still responded to cordycepin (Fig. 9). It is possible that the activation of AMPK is the result of a homeostatic response that resists the effects of cordycepin. This is consistent with the fact that AMPK knockout cells have been reported to be more sensitive to cordycepin [50].

A limitation of our study is that it does not show how cordycepin represses growth factor signalling. Our data are ambiguous as to which pathway, PI3K/AKT/mTOR or MEK/ERK, is directly affected by the target of cordycepin. In the high‐throughput study in NIH3T3 cells, both pathways appear affected (Figs 1 and 2), in the MCF‐7 western blots, MEK appears to be activated rather than inhibited (Fig. 5A, Fig. S1), but the ingenuity pathway analysis finds both MEK inhibitors and PI3K inhibitors as upstream regulators in both MCF‐7 and MDA‐MB‐231 cells (Figs 6 and 7). In contrast, direct comparison of cordycepin to a MEK and a PI3K inhibitor in MCF‐7 cells clearly favours PI3K inhibition as more similar to cordycepin treatment (Fig. 8, Fig. S3). Similarly, the polysome profiling data in Fig. 10 show that effects on mTOR signalling are prominent within 30 min of treatment. Possibly, both pathways are independently affected by cordycepin and the observed effect is dependent on the cell type or the exact experiment. Multiple pathway inhibition could be an advantage in cancer therapy by reducing the chances of the emergence of drug resistance, a common issue in targeted therapy [42, 43, 45].

The effects of cordycepin on immune responses are complex. While we have previously observed that cordycepin represses the inflammatory response [3, 48], several immune response genes are induced by cordycepin in NIH3T3 cells (Fig. 1) and the analysis suggests that genes involved in activation of NK cells are induced. Indeed, NK cell activation by cordycepin has been reported [55]. PI3K/mTOR and MEK/ERK inhibition can affect inflammation and immune responses, and it is therefore likely that the effects on inflammation and immune responses are also mediated by the changes in signal transduction.

Our data on cordycepin metabolism in tissue culture show that cordycepin is taken up by cells and phosphorylated to form cordycepin triphosphate, with levels of cordycepin triphosphate rising to 10 times the medium cordycepin concentration (Fig. 4). Remarkably, a previous study reported an almost complete replacement of ATP with cordycepin triphosphate without immediate loss of cell viability [50]. Inhibition of deaminase enzymes by pentostatin increased the intracellular concentration of cordycepin triphosphate further and enhanced the cytotoxic effects (Figs 3, 4, 5). This indicates that deamination reduces the potency of cordycepin, despite the fact that it can be regenerated [48]. These data support cordycepin triphosphate as the active metabolite of cordycepin. Indeed, the effects of cordycepin on gene expression can be reduced by inhibitors of nucleoside import and phosphorylation [3, 4]. Moreover, a haploid genetic screen showed that mutation of adenosine kinase conveys resistance to cordycepin [22]. The molecular target of cordycepin is therefore likely to bind cordycepin triphosphate.

Cordycepin triphosphate is a proven inhibitor of poly(A) polymerases, which mediate mRNA polyadenylation [9], the final step in making messenger RNAs, while direct effects on transcription are minor [3]. We confirm that cordycepin affects polyadenylation in MCF‐7 cells (Fig. 5D). It is however as yet not clear why inhibiting polyadenylation would lead to effects on growth factor signal transduction. Remarkably, however, a recent study showed that mTOR regulates polyadenylation of ribosomal mRNAs and consequently their translation [56]. It is therefore possible that the effects seen in Fig. 10 are directly by inhibition of polyadenylation.

Our data are supportive of cordycepin as a lead compound for the development of anticancer drugs. The apparent dual action on the two key proliferative pathways, PI3K/AKT/mTOR and MEK/ERK, may account for its strong effects on proliferation and cell survival in tissue culture. However, it appears that deamination reduces its effectiveness. Indeed, NUC7738, a phosphoamidate derivative of cordycepin that evades deamination, is also converted in to cordycepin triphosphate. It is currently in clinical trials [22, 57]. If NUC7738 is proven successful, it will become even more important to elucidate how intracellular cordycepin triphosphate represses growth factor signal transduction.

## Author contributions

SL re‐analysed all the high‐throughput data in this paper and conducted the experiments in Fig. 9 and Fig. S3. JL performed the experiments in Figs 4A, 7, 8 and 10, as well as Figs S1 and S2. AK performed pilot experiments at the very start of this study and contributed Figs 3A and 5B,D and the MCF‐7 microarray experiment in Fig. 6. WU did LC–MS/MS to determine nucleotide and nucleoside levels, as shown in Figs 4 and 5C. RS did pilot experiments for Fig. 8, obtained and quality checked the samples for the microarray experiment in Fig. 1 and the microarray experiment for Fig. 6D. SA performed the experiment in Fig. 3B. GJT and IRM did early analyses of the MCF‐7 experiment that guided our further work. In addition, GJT contributed the analysis in Figs 1B and 6D. KS co‐supervised the bioinformatic work, and D‐HK and DB co‐supervised the analysis of nucleosides and nucleotides. CHM led the work overall and wrote the paper.

### Peer review

The peer review history for this article is available at https://www.webofscience.com/api/gateway/wos/peer‐review/10.1002/1873‐3468.15046.

## Supporting information


**Fig. S1.** Three replicates of the western blots in Fig. 5A.
**Fig. S2.** Cordycepin affects some growth factor regulated genes in MBA‐MB468 breast cancer cells.
**Fig. S3.** Comparison between cordycepin and kinase modulators.
**Fig. S4.** AMPK knockout alters but does not prevent the response to EGF in HEK293 cells.
**Fig. S5.** Gene ontology analysis of genes with increased polysomal association.
**Fig. S6.** Multidimensional scaling (MDS) plot of HEK293 RNA‐Seq biological replicates.

**Table S1.** Primer sequences for RT‐qPCR experiments.

## Data Availability

Data have been deposited in the GEO database under the following numbers: GSE277557 (NIH3T3), GSE277620 (MCF‐7 cordycepin treatment), GSE277763 (MCF‐7 serum withdrawal), GSE277827 (MDA‐MB‐231), GSE277825 (HEK293) and GSE277765 (MCF‐7 polysome profiling). The RAW264.7 data reused in Fig. 11 were deposited previously as GSE126157.
