# Supplementary material for: Cordycepin generally inhibits growth factor signal transduction in a systems pharmacology study
Source: FEBS Lett. 2024 Nov 7;599(3):415–35. doi: 10.1002/1873-3468.15046 (PMC11808429; doi:10.1002/1873-3468.15046)
Supplement: Supplementary file 1 — Fig. S1. Three replicates of the western blots in Fig. 5A. Fig. S2. Cordycepin affects some growth factor regulated genes in MBA‐MB468 breast cancer cells. Fig. S3. Comparison between cordycepin and kinase modulators. Fig. S4. AMPK knockout alters but does not prevent the response to EGF in HEK293 cells. Fig. S5. Gene ontology analysis of genes with increased polysomal association. Fig. S6. Multidimensional scaling (MDS) plot of HEK293 RNA‐Seq biological replicates. [file FEB2-599-415-s002.pdf]

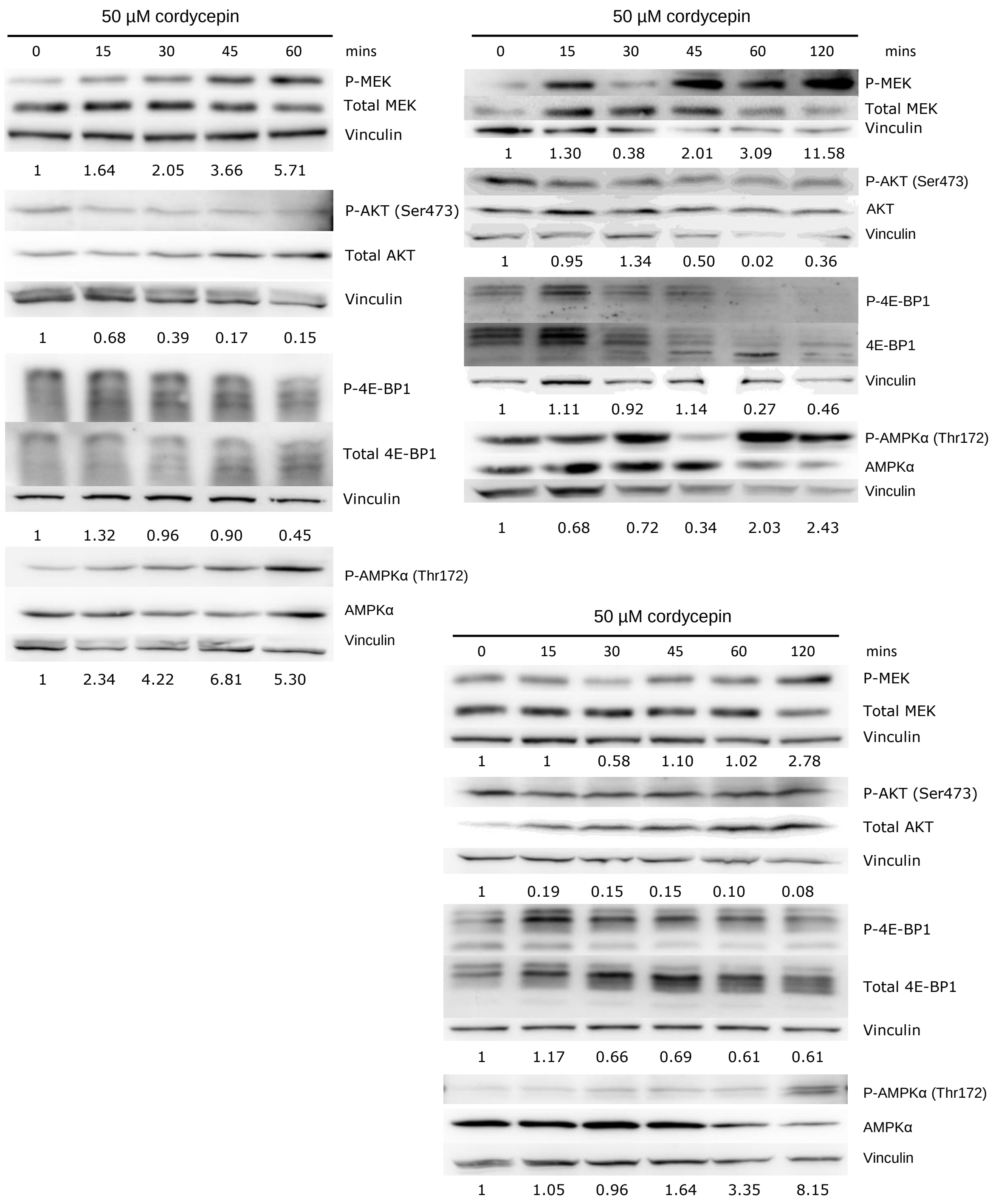

Supplementary Figure 1. **Three replicates of the western blots in Figure 5A.** Western blots of cells treated with 50 µM cordycepin for the indicated times stained with the indicated antibodies. Vinculin: loading control. Numbers below the blots indicate the ratio between phosphorylated and total signal.

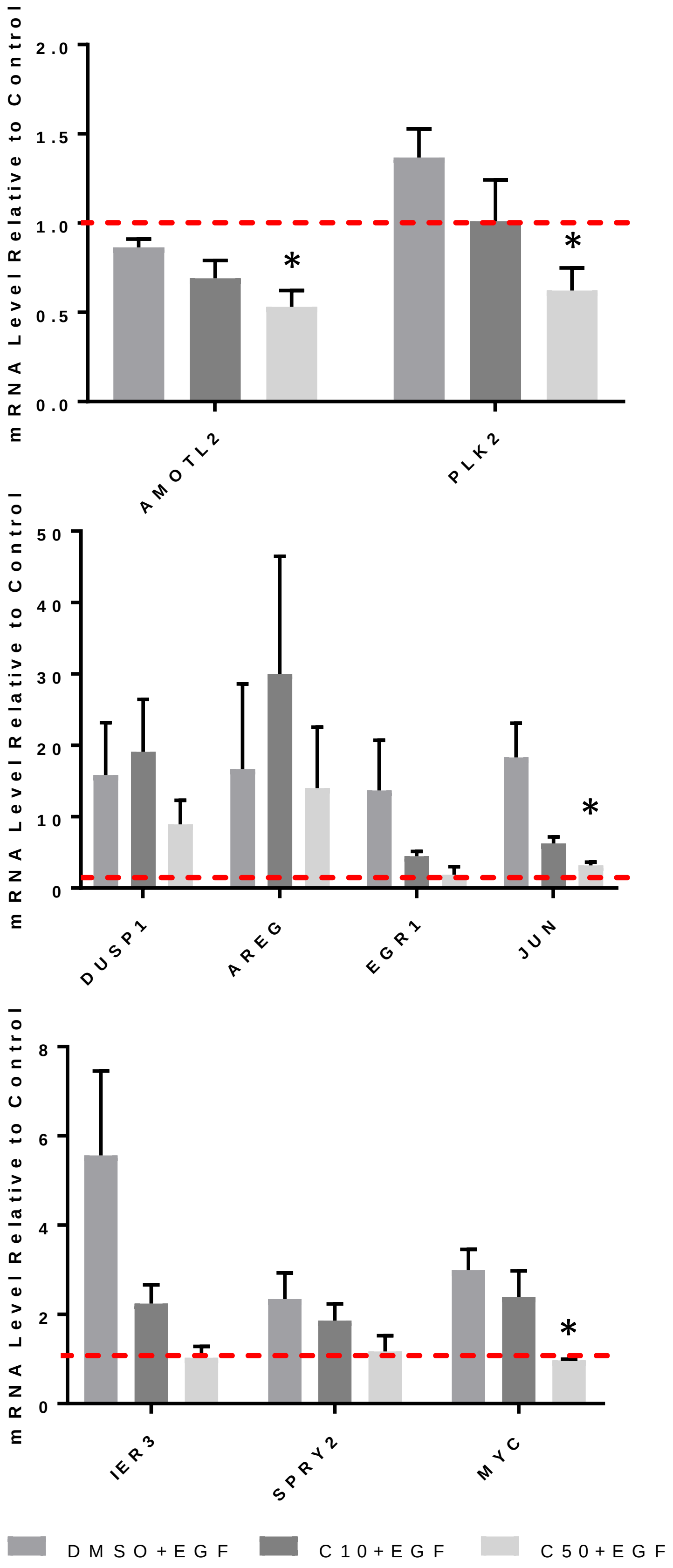

Supplementary Figure 2. **Cordycepin affects some growth factor regulated genes in MBA-MB468 breast cancer cells.** MDA-MB-468 cells were treated with EGF 1 hr before collection. Cordycepin of different concentrations were administered 30 mins before collection. Fold change mRNA levels relative to no EGF+DMSO control (dashed line) were determined with RT-qPCR with GAPDH as the reference gene. Ct values of GAPDH. (mean ± SD, n = 3 independent experiments, *P<0.05 (Dunnett's test). Fold change of the mRNA level caused by EGF stimulation, and EGF with cordycepin at a final concentration of 10 µM (C10) or 50 µM (C50).

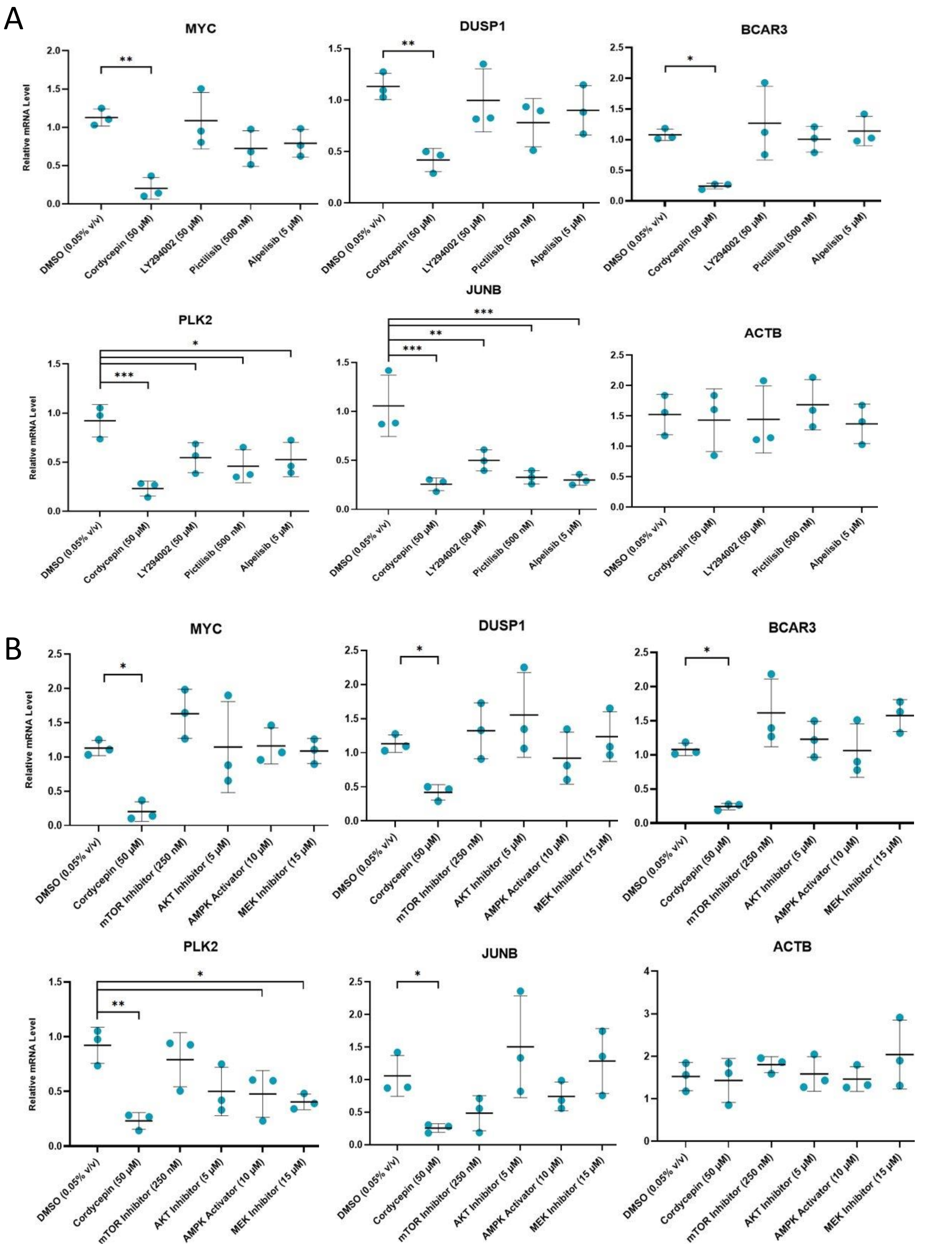

Supplementary Figure 3. **Comparison between cordycepin and kinase modulators.** MCF-7 breast adenocarcinomas were treated for 2 hours with DMSO (0.05% v/v) and cordycepin (50 µM) and different signal transduction modulators. Relative mRNA expression level of tested genes by RT-qPCR are presented relative to untreated control. A. PI3K inhibitors LY294002 (50 µM), Pictilisib (500 nM), or Alpelisib (5 µM). B. Protein kinase modulators Torin1 (500 nM; mTOR inhibitor), MK-2206 (5 µM; AKT inhibitor), A-769662 (10 µM; AMPK activator), or PD98059 (15 µM; MEK inhibitor). Graphed is mean ± SD; n=3 independent experiments; One-way Anova was used to obtain statistical significance against DMSO (0.05% v/v): *P<0.05, **P<0.01, ***P<0.001).

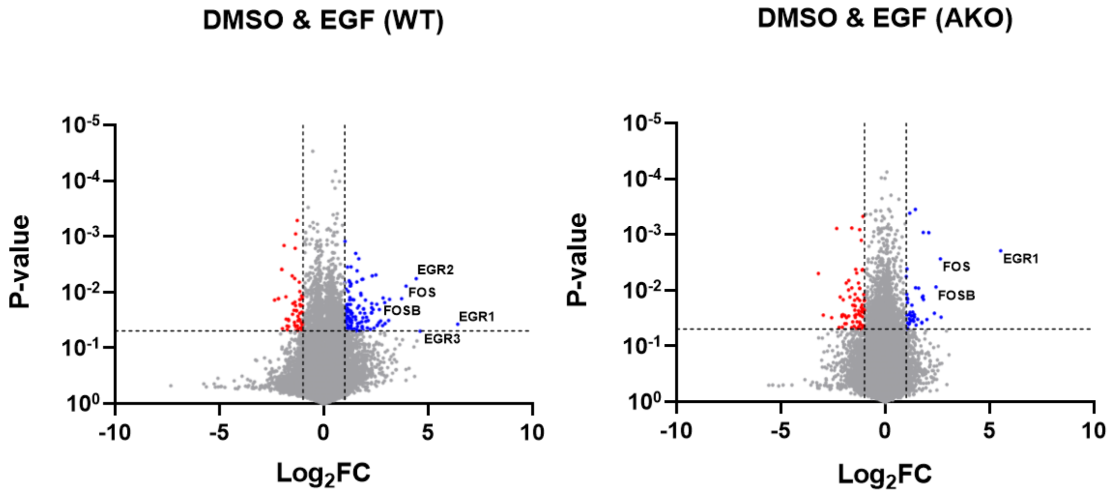

Supplementary Figure 4. **AMPK knockout alters but does not prevent the response to EGF in HEK293 cells.** AMPK CRISPR-Cas9 knockout and wild type HEK293 cells were serum starved for 24 hours prior to treatment with DMSO (0.025% v/v) for 20 minutes before stimulation with EGF (15 nM) for 30 minutes, or DMSO (0.025% v/v) on its own. Total RNA was extracted and sent off for RNA-Seq with output analysed through Log2 fold change of treatment versus control of RPKM values after Upper Quartile normalisation. Each dot represents a differentially expressed gene with DMSO (0.025% v/v) and EGF stimulation. Red denotes downregulated genes with ≤ -1 Log2FC & ≤ 0.05 p-value, blue denotes upregulated genes with ≥ 1 Log2FC & ≤ 0.05 p-value, grey denotes genes which do not meet these requirements

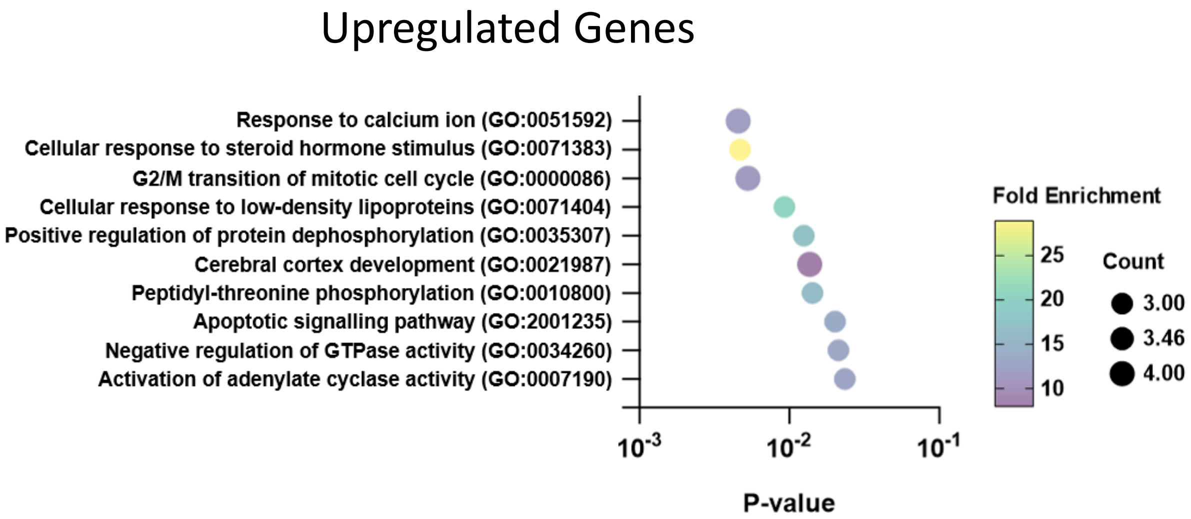

Supplementary Figure 5. **Gene ontology analysis of genes with increased polysomal association.** Polysome profliling of lysates from MCF-7 cells treated for 30 minutes with DMSO (control) or 50 µM cordycepin. Gene ontology analysis of the upregulated genes.

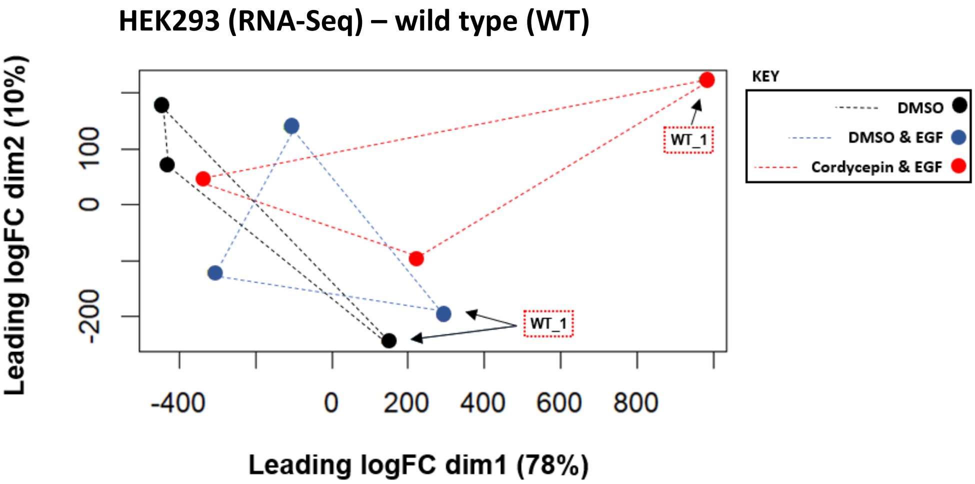

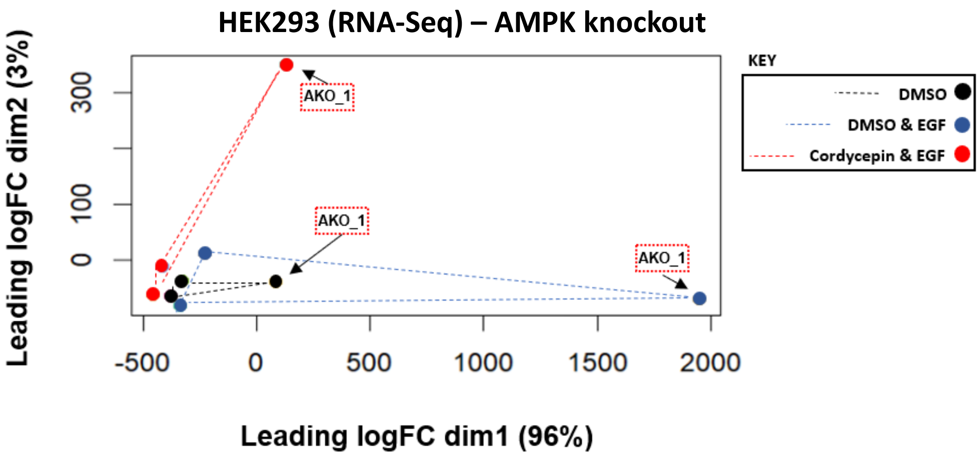

Supplementary Figure 6. **Multidimensional scaling (MDS) plot of HEK293 RNA-Seq biological replicates.** RPKM values of HEK293 RNA-Seq samples were used for MDS using the ‘plotMDS’ function within the LIMMA tool(686). The distances between the plots correlates to the dissimilarity between the expression (Log2FC) of the top 500 genes. The X- and Y-axis represent the Euclidean distances (difference between two plots). The dimensions (dim1 and dim2) explain the percentage of total variance between the expression of the samples. The dotted lines show the spread of expression between biological replicates of treatment conditions to show outliers in the replicates. Replicate 1 was deemed an outlier and removed from analysis.
