# Supplementary material for: Cordycepin generally inhibits growth factor signal transduction in a systems pharmacology study
Source: FEBS Lett. 2024 Nov 7;599(3):415–35. doi: 10.1002/1873-3468.15046 (PMC11808429; doi:10.1002/1873-3468.15046)
Supplement: Supplementary file 2 — Table S1. Primer sequences for RT‐qPCR experiments. [file FEB2-599-415-s001.pdf]

Supplementary Table

gene	Orientation	Primer Sequence (5'-3')	Species
ACTB	Forward	AACCGCGAGAAGATG	*Homo sapiens*
ACTB	Reverse	CCAGAGGCGTACAGGGATAG	*Homo sapiens*
AMOTL2	Forward	CCATGAGAGCCTGACCAGAG	*Homo sapiens*
AMOTL2	Reverse	GCCTCCTGTGTCTTGCTTG	*Homo sapiens*
AREG	Forward	TTGATACTCGGCTCAGGCCA	*Homo sapiens*
AREG	Reverse	GTAGTCATAGTCGGCTCCCG	*Homo sapiens*
ATF3	Forward	CACAAAAGCCGAGGTAGC	*Homo sapiens*
ATF3	Reverse	AGCCTTCAGTTCAGCATTCAC	*Homo sapiens*
BCAR3	Forward	GGCACCAGTACACCCAAACT	*Homo sapiens*
BCAR3	Reverse	CACATGTCGGTTCCTTCAAA	*Homo sapiens*
CTGF	Forward	CAGAGCAGCTGCAAGTACCA	*Homo sapiens*
CTGF	Reverse	GCCAAACGTGTCTTCCAGTC	*Homo sapiens*
DUSP1	Forward	GAGCTGTGCAGCAAACAGT	*Homo sapiens*
DUSP1	Reverse	CAGGTACAGAAAGGGCAGGA	*Homo sapiens*
EEF1A2	Forward	GTCAAGGAAGTCAGCGCCTA	*Homo sapiens*
EEF1A2	Reverse	CTTGAACCACGGCATGTTGG	*Homo sapiens*
EGR1	Forward	TTCACGTCTTGGTGCCTTTT	*Homo sapiens*
EGR1	Reverse	AGCGGCCAGTATAGGTGAT	*Homo sapiens*
FOS	Forward	CTGTCAACGCGCAGGACTT	*Homo sapiens*
FOS	Reverse	GTCATGGTCTTCACAACGCC	*Homo sapiens*
FOSL1	Forward	CAGGCGGAGACTGACAAAC	*Homo sapiens*
FOSL1	Reverse	CCTGGGGAAAGGGAGATACA	*Homo sapiens*
GAPDH	Forward	CATCGCTCAGACACCATGGG	*Homo sapiens*
GAPDH	Reverse	CGTTCTCAGCCTTGACGGTG	*Homo sapiens*
IER3	Forward	GGCTTCTCTTTCTGCTGCTC	*Homo sapiens*
IER3	Reverse	ACACCCTCTTCAGCCATCAG	*Homo sapiens*
JUN	Forward	CGTGAAGTGACGGACTGTTC	*Homo sapiens*
JUN	Reverse	GTGAGGAGGTCCGAGTTCTT	*Homo sapiens*
JUN	Forward	ACAGAGCATGACCCTGAAAC	*Homo sapiens*
JUN	Reverse	CCGTTGCTGGACTGGATTAT	*Homo sapiens*
JUNB	Forward	AAGGGACACGCCTTCTGAA	*Homo sapiens*
JUNB	Reverse	AAACGTCGAGGTGGAAGGA	*Homo sapiens*
MCL1	Forward	AGAAAGCTGCATCGAACCA	*Homo sapiens*
MCL1	Reverse	CCAGCTCCTACTCCAGCAA	*Homo sapiens*
MYC	Forward	TCCTCGGATTCTCTGCTCTC	*Homo sapiens*
MYC	Reverse	CTCTGACCTTTTGCCAGGAG	*Homo sapiens*
NFKB1A	Forward	GCTCCGAGACTTTCGAGGAA	*Homo sapiens*
NFKB1A	Reverse	CAGGGCTCCTGAGCATTGA	*Homo sapiens*
PLK2	Forward	AAAGGTGTTGACAGAGCCAGA	*Homo sapiens*
PLK2	Reverse	AGACCGAAGTCCCCAACTTT	*Homo sapiens*
PLK2	Forward	GGTGCATAAGGGAAGCAAG	*Homo sapiens*
PLK2	Reverse	AGTCTGTCCGGAGTGAAGC	*Homo sapiens*
RPL10A	Forward	TCTCTCGCGACACCCTGT	*Homo sapiens*
RPL10A	Reverse	TTAGCCTCGTCACAGTGCTG	*Homo sapiens*
RPL23A	Forward	CCTGATTCGGCCTGATGGAG	*Homo sapiens*
RPL23A	Reverse	CTCCCAGCCCAACCAGAAAT	*Homo sapiens*
RPL7	Forward	GAATGGCGAGGATGGCAAGA	*Homo sapiens*
RPL7	Reverse	GGGCTCACTCCATTGATACCTCT	*Homo sapiens*
RPS15A	Forward	CGCGCCGCCACAATG	*Homo sapiens*
RPS15A	Reverse	CGGACGATGACTTTGGAGCA	*Homo sapiens*
RPS18	Forward	CACTGAGGATGAGGTGGAACG	*Homo sapiens*
RPS18	Reverse	GAAGTGACGCAGCCCTCTAT	*Homo sapiens*
SGK1	Forward	ATGAAGCAGAGGAGGATGGG	*Homo sapiens*
SGK1	Reverse	GGCCAAGGTTGATTTGCTGA	*Homo sapiens*
SPRY2	Forward	GCGCTTGTAGAAGGGGAGTC	*Homo sapiens*
SPRY2	Reverse	ACACATCTGAACTCCGTGATCG	*Homo sapiens*
WEE1	Forward	GGGAATTGATTCCAGCTCT	*Homo sapiens*
WEE1	Reverse	CACTGGCTTCCATGTCTTCA	*Homo sapiens*
Act1	Forward	CCGGACTCGAGAAGAAACAT	*Schizosaccharomyces pombe*
Act1	Reverse	AACCACCTTTTTCCGCTCTT	*Schizosaccharomyces pombe*

Supplementary Table 1. **Primer sequences for RT-qPCR experiments**
